# Synthesis and Antiplasmodial Activity of 1,2,3-Triazole-Naphthoquinone Conjugates

**DOI:** 10.3390/molecules24213917

**Published:** 2019-10-30

**Authors:** Sandra Oramas-Royo, Priscila López-Rojas, Ángel Amesty, David Gutiérrez, Ninoska Flores, Patricia Martín-Rodríguez, Leandro Fernández-Pérez, Ana Estévez-Braun

**Affiliations:** 1 Instituto Universitario de Bio-Orgánica (CIBICAN), Departamento de Química Orgánica, Universidad de La Laguna, 38206 Tenerife, Spain; priscila.quim@gmail.com; 2 Instituto de Investigaciones Fármaco Bioquímicas, Facultad de Ciencias Farmacéuticas y Bioquímicas, Universidad Mayor de San Andrés, Av. Saavedra 2024, 2° piso, Miraflores, La Paz 2314, Bolivia; doyneldavid@yahoo.es (D.G.); ninofq@hotmail.com (N.F.); 3 Instituto Universitario de Investigaciones Biomédicas y Sanitarias (IUIBS), Departamento de Ciencias Clínicas, BIOPHARM, Universidad de Las Palmas de Gran Canaria, 35001 Las Palmas de Gran Canaria, Spain; patrimar_bio@hotmail.com (P.M.-R.); leandrofco.fernandez@ulpgc.es (L.F.-P.)

**Keywords:** malaria, *Plasmodium falciparum*, 1,2,3-triazole-naphthoquinones, copper-catalyzed cycloaddition, docking

## Abstract

A series of 34 1,2,3-triazole-naphthoquinone conjugates were synthesized via copper-catalyzed cycloaddition (CuAAC). They were evaluated for their in vitro antimalarial activity against chloroquine-sensitive strains of *Plasmodium falciparum* and against three different tumor cell lines (SKBr-3, MCF-7, HEL). The most active antimalarial compounds showed a low antiproliferative activity. Simplified analogues were also obtained and some structure–activity relationships were outlined. The best activity was obtained by compounds **3s** and **3j,** having IC_50_ of 0.8 and 1.2 μM, respectively. Molecular dockings were also carried on *Plasmodium falciparum* enzyme dihydroorotate dehydrogenase (*Pf*DHODH) in order to rationalize the results.

## 1. Introduction

Malaria, a disease caused by parasites of the *Plasmodium* genus and spread through the bites of infected mosquitoes, was responsible for an estimated 219 million clinical cases and 435,000 deaths worldwide in 2017, mostly among children under the age of five [1]. Most of the malarial infections and deaths are due to *Plasmodium falciparum* and *Plasmodium vivax* species. Available therapeutic agents are already limited in their efficacy, and drug resistance threatens to diminish the ability to treat and prevent the disease further. Despite a renewed effort to identify antimalarial compounds, the drug discovery lacks target diversity and most malaria drugs are only efficacious during the asexual blood stage of parasite infection [2]. Thus, it is necessary the search for new antimalarial drugs that overcome the resistance and act through new mechanisms.

Natural products have played a pivotal role in the discovery of lead compounds for the treatment of malaria, from quinine and artemisinin to ozonide-based compounds. Many of these natural products have served as a starting point for the development and design of antimalarial drugs currently in the clinic or in the development phase [3].

1,4-Naphthoquinones are a class of compounds broadly studied in natural products chemistry and medicinal chemistry [4,5,6]. Within these studies, the antimalarial activity has been reported for several 1,4-naphthoquinones [7,8,9,10,11,12,13,14]. The antiparasitic activity of the quinones is related to several mechanisms such as enzymatic inhibition (e.g., dihydroorotate dehydrogenase, glutathione reductase, and glycerol glyceraldehyde-3-phosphate dehydrogenase), alkylation of biomolecules, competitive inhibition of the cytochrome bc1 complex, depletion of glutathione, and generation of reactive oxygen species, among others [15]. Lawsone derivatives containing the 2-hydroxy-1,4-naphthoquinone scaffold have been highly explored for the development of novel antiparasitics [7,16,17,18,19]. Atovaquone, buparvaquone, and parvaquone and are 3-substituted-2-hydroxy-1,4-naphthoquinones used as antipneumocystic (buparvaquone and parvaquone) and antimalarial (atovaquone) agents, which exemplifies the potentiality of such scaffold in the development of novel drugs [3]. Additionally, some studies indicate the potential application of natural or synthetic 2-amino-1,4-naphthoquinones and 2-amino-1,2-naphthoquinones as antiplasmodial agents [20].

Nowadays, molecular hybridization has emerged as a promising tool for the drug design process and medicinal chemistry. In this strategy, two or more different pharmacophoric units are covalently linked into a single hybrid molecule with best properties as compared to the parent drugs [20]. Molecular hybridization [21] is beneficial, as different targets are activated by a single molecule, and is particularly interesting where treatment is limited to a few commercial drugs or in cases where the bioactive compounds present pharmacokinetic and pharmacodynamic limitations or high toxicity.

**Figure 1:**
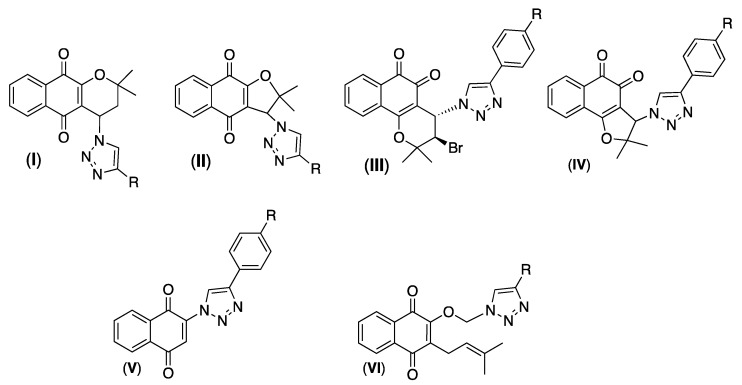
Structures of antiparasitic naphtoquinone triazoles.

Guided by the concept of molecular hybridization, some triazole naphthoquinones have been prepared as trypanocidal agents. Thus, da Silva Júnior et al. synthesized diverse α-lapachone-triazoles (**I**) [22], nor-α-lapachone-triazoles (**II**) [22,23], β-lapachone-triazoles (**III**) [24], nor-β-lapachone-triazoles (**IV**) [25,26], and 1,4-naphthoquinone coupled 1,2,3-triazoles (**V**) [25] (Figure 1), some of them resulted in being potent compounds against antimony-sensitive and -resistant leishmania.

Recently, Pereira et al. have reported the antimalarial activity against *Plasmodium falciparum* (W2) of a set of triazole-lapachol derivatives (**VI**) with values of IC_50_ in the range of 4.5–197.7 μM [27].

In the present work a new series of naphthoquinonolyl triazole derivatives have been synthesized from lawsone (2-hydroxy-1,4-naphthoquinone) and evaluated for their *in vitro* activity against chloroquine-sensitive strains of *Plasmodium falciparum* and for antiproliferative activity against some tumor cell lines. From the obtained results some structure–activity relationships were outlined. Furthermore, docking studies on the *Plasmodium falciparum* enzyme dihydroorotate dehydrogenase (*Pf*DHODH) suggested that these compounds could act as inhibitors of this enzyme.

## 2. Results and Discussion

The 1,2,3-triazole-naphthoquinone derivatives (**3**) were synthesized using a copper(I)-catalyzed Huisgen 1,3-dipolar cycloaddition reaction [28] of the corresponding *O*-propargylated naphthoquinone (**1**) with alkyl or aryl azides (**2**) (Table 1). 

The starting *O*-propargyl-naphthoquinone was prepared in good yields by reaction of 2-hydroxy-1,4-naphthoquinone and propargyl bromide using K_2_CO_3_ in dimethylformamide. The aryl and alkyl azides were obtained from alkyl bromide or boronic acids and sodium azide [29].

Table 1 shows the structures and yields of a variety of substituted 1,2,3-triazole-1,4-naphtho quinone conjugates. As we can see, moderate-to-good yields were obtained. The structures of compounds (**3a**–**3u**) were characterized by ^1^H-NMR, ^13^C-NMR, and HRMS. In the ^1^H-NMR, the triazolic hydrogen appeared as a singlet at δ 8–10 ppm, the naphthoquinone hydrogen and the methylene hydrogens were also observed as singlets at δ 6.3–6.7 ppm and δ 5.1–5.5 ppm, respectively. Conjugates with aliphatic substituents on the triazol moiety were prepared (**3a**–**3c**, **3f**, **3o**). Some derivatives containing different electron donating and withdrawing groups linked to the phenyl group of the triazole ring were also obtained (**3j**–**3s**). Finally, diverse hybrid compounds were prepared using the triazole ring as a linker between the naphthoquinone nucleus and diverse privileged structures such as coumarin (**3g**), 1,8-naphthalimide (**3h**–**3i**), 1,4-naphthoquinone (**3d**–**3e**), and indol (**3t**).

The obtained 1,2,3-triazole-naphthoquinone derivatives (**3a**–**3u**) were evaluated in vitro against F-32 Tanzania (chloroquine sensitive) strains of *Plasmodium falciparum*. These compounds were also tested against SKBr-3 (human breast cancer), MCF-7 (human breast adenocarcinoma), and HEL (human erythroleukemia) tumor cell lines. The results obtained are shown in Table 2.

From the obtained results some structure–activity relationships were outlined. Regarding the antiplasmodial activity, 16 compounds of the 21 were active with IC_50_ values ranging from 17.9 to 0.8 μM. The introduction of indol (**3t**), coumarin (**3g**), N-substituted-naphthalimide (**3h**), and 2-amine-1,4-naphthoquinone (**3e**) on the 1,2,3-triazole ring led to inactive compounds, whereas the N-unsubstituted naphthalimide (**3d**) and the 1,4-naphthoquinone (**3d**) had IC_50_ values of 5.5 ± 0.1 and 1.9 ± 0.09 μM, respectively. The conjugate derivatives with alkyl chains (**3a**–**3b**) and benzyl group (**3c**) on the triazole ring showed worse activities than those with an attached aryl group. In the aryl series (**3j**–**3s**), similar antiplasmodial activity was observed with electron-donating (**3k**, **3l**, **3o**) or electron-withdrawing substituents in the aromatic ring. In fact, the best IC_50_ values were achieved by compound **3s** (0.8 ± 0.1 μM) bearing a methoxy at C-4 and a fluor group at C-3 and **3j** (1.2 ± 0.2 μM) with an unsubstituted phenyl ring. The presence of hydrogen bond donors such as -CH_2_OH (**3p**) or -SH (**3q**) produced inactivity. The introduction of conjugated systems such as [1,1′-biphenyl]-4-carbonyl (**3f**) or 1-(trifluoromethyl)-4-vinylbenzene (**3u**) led to good values of IC_50_ (1.6 ± 0.1 and 3.3 ± 0.07 μM, respectively).

Concerning the antiproliferative activity most part of the compounds showed IC_50_ > 10 μM against the three tumor cell lines assayed. Only compounds **3a**, **3c**, **3h**, **3i**, **3p**, and **3r** presented moderate antiproliferative activity against HEL (human erythroleukemia) with IC_50_ values ranging from 3.8 to 7.3 μM. These results are interesting from a selective index point of view, as the most active antiplasmodial compounds (**3d**, **3f**, **3j**, **3l**, **3m** and **3s**) present a low antiproliferative activity.

**Scheme 1:**
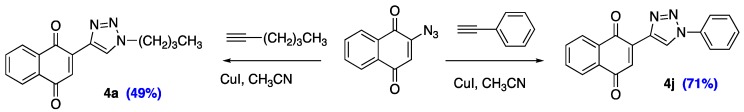
Synthesis of compounds **4a** and **4j**.

In order to deepen the structural determinants responsible of the antiplasmodial activity, we decided to prepare several derivatives. Thus, we wanted to evaluate the influence in the activity of the -CH_2_O- linker, and two compounds (**4a** and **4j**) were synthesized with the triazol ring attached at carbon C-2 of the naphthoquinone nucleus (Scheme 1).

**Figure 2:**
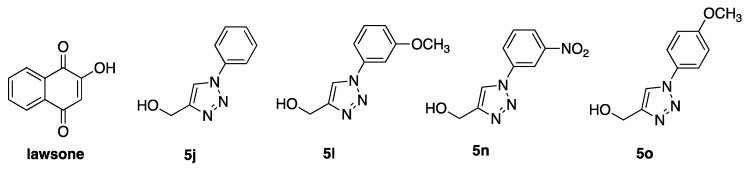
Structures of simplified fragments of 1,2,3-triazole-naphthoquinones.

When **4a** and **4j** were assayed, a loss of activity (IC_50_ > 10 μg/mL) with respect to the active compounds **3a** and **3j**, having the -OCH_2_- linker, was detected. Next, we analysed the antiplasmodial activity of the 2-hydroxy-1,4-naphthoquinone (lawsone) and some (4-aryl-1*H*-1,2,3-triazol-1-yl) methanol derivatives (Figure 2 and Supplementary Material) as simplified fragments of the active structures. All of them were inactive (IC_50_ > 10 μg/mL), which indicated the importance for the biological activity of both the quinone ring and the substituted triazole.

Next, we evaluated the influence on the activity of an isosteric modification by replacing the -OCH_2_- linker by -NHCH_2_-. Thus, the following conjugates were prepared from N-propargylated-1,4-naphthoquinone and alkyl and aryl azides in higher yields than those from *O*-propargylated-1,4-naphthoquinone (Table 3). None of the N-derivatives resulted active (IC_50_ > 10 μg/mL), which indicated that the replacing of an acceptor of hydrogen bonds by donor hydrogen bonds in this part of the molecule led to a drastic loss of activity.

In order to rationalize all these results, we carried out molecular dockings on the enzyme dihydroorotate dehydrogenase (DHODH). Dihydroorotate dehydrogenase (DHODH) is an enzyme essential to the fourth and rate limiting step in de novo pyrimidine biosynthesis, and it catalyzes the conversion of dihydroorotate (DHO) to orotate (ORO) with the reduction of ubiquinone. The significance of pyrimidine bases for cell proliferation and metabolism determines human DHODH as an attractive target for the development of new drug candidates in different clinical applications for arthritis, malaria, and cancer [30,31].

*Plasmodium falciparum*, the major human malarial parasite, is particularly susceptible to DHODH inhibition because the *P. falciparum* is dependent on de novo pyrimidine biosynthesis, at least during the parasite’s intraerytrocytic stage [32]. The inhibitor-binding site of P*f*DHODH, located in proximity to the cofactor-binding site, is characterized by the presence of two regions—the H-bond pocket, comprising His 185, Tyr 528, and Arg 265, and the hydrophobic pocket. The size of the hydrophobic pocket is variable, depending on the conformations of the side chains of Phe 171 and Phe 188. In addition, it is observed that Met 536 and Tyr 168 are two additional residues with a high degree of conformational flexibility in the same hydrophobic pocket [33]. Several computational studies have been done using various approaches with the goal of finding P*f*DHODH inhibitors [34,35]. On the basis of this information and in order to understand the probable binding mode and to propose a mode of action of the antiplasmodial activity of synthesized 1,2,3-triazole-naphthoquinone conjugates, a molecular docking study was performed on reported crystal structure of *Plasmodium falciparum* enzyme dihydroorotate dehydrogenase using the Glide software [36]. The X-ray crystal structure of P*f*DHODH (Protein Data Bank (PDB) 1TV5) [37] was retrieved from the Protein Data Bank having a resolution of 2.4 Å, which has been reported to be used frequently for docking studies [38]. Hence, we docked all the 1,2,3-triazole-naphthoquinone conjugates into the putative quinone-binding tunnel formed by the N-terminal domain to see if these compounds could interact with this target and, consequently, to understand the possible key active site interactions.

**Figure 3:**
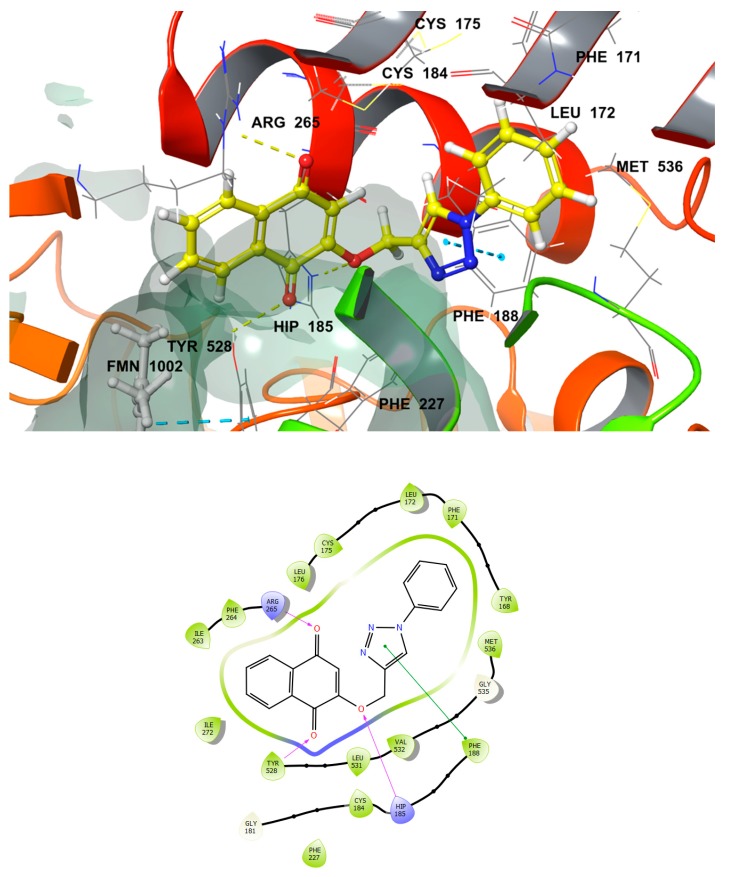
Binding mode prediction of **3j** with *Plasmodium falciparum* enzyme dihydroorotate dehydrogenase (PfDHODH) (Protein Data Bank (PDB) code 1TV5).

An analysis of the docking results clearly indicated that one of the most active compounds in this study, **3j**, showed substantial binding affinities, a good steric and electronic complementarity, and fits well into the P*f*DHODH binding site. In addition, these docking results strongly suggested that the compound **3j** and the inhibitor bounded in the putative quinone-binding tunnel shared a common binding mode. The best docking score was −6.70 kcal mol^−1^. According to the predicted binding modes, the compound **3j** showed a π–π stacking interaction between the triazol ring present in these compounds with the amino acid residue Phe 188 (Figure 3).

In addition, three hydrogen bonds were detected, two of them between Arg 265 and Tyr 528 with the carbonyl groups belonging to the quinone moiety and another hydrogen bond between His 185 and the ether group that link the quinone moity and the triazole ring. Moreover, in the favored docking conformation, many hydrophobic side chain residues of the putative quinone-binding tunnel were in close proximity to naphthoquinone-triazole conjugates. In the predicted pose of the compound **3j**, the potential hydrophobic interactions involve residues such as Cys 175, Cys 184, Phe 227, Leu 172, Met 536, and Phe 171. The presence of polar contacts with a considerable number of residues, as well as hydrophobic interactions, seem to play a fundamental role in the binding of these compounds, which could explain their antiplasmodial activity.

Finally, some physicochemical descriptors (MW, LogP, H-bond donors, H-bond acceptors, rotable bonds, and TPSA) for the best active compounds were calculated using Molinspiration Cheminformatics software (2019) and the corresponding values are included in Table 4. As we can observe, all of them showed values within the accepted ranges for drug-like molecules.

## 3. Materials and Methods

### 3.1. General Methods

The reactions under microwave irradiation were performed in a Biotage Initiator 2.5 using standard sealed microwave glass vials (2–5 mL) and a normal absorption level. Solvents were dried immediately prior to use by distillation from a drying agent: Tetrahydrofuran (THF) from Na/benzophenone and CH_3_CN from CaH_2_ [39]. Commercial reagents were purchased from Sigma-Aldrich Chemical Co. and Alfa Aesar and were used without further purification. Analytical thin-layer chromatography was performed on Polygram SIL G/UV254 silica gel plates and chromatograms were visualized under UV light (254 and 360 nm). Purification on column chromatography was carried out on Merck silica gel 60 (0.063–0.2 mm) with the indicated solvent mixtures. Pre-coated TLC plates SIL G-100 UV254 (Macherey–Nagel) and SILICA GEL GF plates (1000 μm, Analtech) were used for preparative TLC purification. ^1^H and ^13^C-NMR spectra were acquired in CDCl_3_ (0.03% *v*/*v* TMS), DMSO-*d*_6_ or CD_3_CN at room temperature using Bruker Avance instruments (500 or 600 MHz for ^1^H-NMR and 125 or 150 MHz for ^13^C-NMR). Chemical shifts were reported in parts per million (ppm) from tetramethylsilane and referenced to the residual solvent peak (CDCl_3_: δ 7.26 for ^1^H-NMR, δ 77.00/77.16 for ^13^C-NMR; DMSO-*d*_6_: δ 2.50 for ^1^H-NMR, δ 39.52 for ^13^C-NMR; CD_3_CN: δ 1.93 for ^1^H-NMR, δ 1.32, 118.26 for ^13^C-NMR). For ^1^H-NMR, data were reported in the following manner: chemical shift (integration, multiplicity, coupling constant where applicable). The following abbreviations were used: s (singlet), br (broad), d (doublet), t (triplet), dd (double doublet), td (triplet of doublets), m (multiplet). Coupling constants (*J*) were given in Hertz (Hz). ^13^C-NMR were obtained with complete proton decoupling. MS and HRMS data were recorded in a VG Micromass ZAB-2F spectrometer and an ESI instrument LCT Premier XE Micromass (ESI-TOF). IR spectra were recorded on a Bruker IFS 28/55 spectrophotometer. All compounds were named using the ACD40 Name-Pro program, which is based on IUPAC rules.

### 3.2. General Procedures for the Synthesis of 4-Substituted 1,2,3-Triazolyl-Naphthoquinonic Derivatives

*Method A:* A solution of 0.57 mmol of the corresponding azide and 121.9 mg (0.57 mmol) of the alkyne in 6 mL of CH_2_Cl_2_ (DCM) was added to a mixture of CuSO_4_**^.^**5H_2_O (5.7 mg, 0.023 mmol) and sodium ascorbate (13.7 mg, 0.069 mmol) in 6 mL of water. The reaction mixture was stirred vigorously at room temperature until the disappearance of the alkyne. Then it was extracted with DCM (3 × 15 mL) and purified through silica gel column chromatography with a growing gradient of polarity DCM:MeOH (100:0–0:100).

*Method B:* The alkyne and 1.0 equivalent of the corresponding azide were dissolved in 5 mL of dried CH_3_CN. Then, CuI (15–25 mol %) was added and the reaction mixture was stirred under argon atmosphere and at room temperature for 72 h. The solvent was removed under reduced pressure and purified through silica gel column chromatography with a growing gradient of polarity DCM:MeOH (100:0–0:100) when needed.

*Method C:* A solution of the corresponding boronic acid (0.24 mmol) and 78.5 mg (1.2 mmol) of sodium azide in 1.5 mL of H_2_O were added to a vigorously stirred mixture of Cu_2_O (3.4 mg, 0.024 mmol) in 0.06 mL of 20% of NH_3_ and 0.12 mL of H_2_O. The reaction mixture was stirred for 16 h at room temperature under an oxygen atmosphere. Then, 30 mg (0.14 mmol) of *N*-propargylated naphthoquinone (**6**), 8.11 mg (0.041 mmol) of sodium ascorbate, 1.5 mL of H_2_O, and 3 mL of acetone were added. After stirring for 48 h, the reaction mixture was extracted with ethyl acetate (EtOAc). Subsequently, the aqueous phase was acidified with 5% HCl until pH 2 and it was extracted with EtOAc (3 × 15 mL). The combined organic phases were dried over anhydrous MgSO_4_ and, after the elimination of the solvent, the corresponding residue was purified by silica gel column cromatography (CC) or TLC-preparative with DCM or 5% DCM/MeOH.

### 3.3. 2-[(1-Butyl-triazol-4-yl)methoxy]-naphthalene-1,4-dione (**3a**)

A variation of method A was used for the synthesis of this compound. As 1-azidobutane is very volatile, we obtained better results when it was synthesized in situ by dissolving 101.4 µL (0.94 mmol) of 1-bromo-butane and 306.8 mg (4.7 mmol) of NaN_3_ in 8 mL of dimethyl formamide (DMF) under an argon atmosphere. Then, the reaction mixture was refluxed for 24 h. After it reached room temperature, 100.0 mg (0.47 mmol) of the alkyne, 4.7 mg (0.019 mmol) of CuSO_4_**^.^**5H_2_O, 11.2 mg (0.057 mmol) of sodium ascorbate, and 8 mL of water was added. After 48 h, the reaction mixture was extracted with EtOAc (3 × 15 mL) and the combined organic phases were dried over anhydrous MgSO_4_ and after elimination of the solvent, and purified through silica gel column chromatography with a growing gradient of polarity DCM:MeOH (100:0–0:100). Hence, 97.2 mg (54%) of compound **3a** was obtained. ^1^H-NMR (CDCl_3_, 400 MHz) δ 8.04 (2H, m), 7.73 (1H, s), 7.71 (2H, m), 6.38 (1H, s), 5.22 (2H, s), 4.34 (2H, t, *J* = 7.2 Hz), 1.87 (2H, m), 1.33 (2H, m), 0.92 (3H, t, *J* = 7.3 Hz); ^13^C-NMR (CDCl_3_, 100 MHz) δ 184.8 (C), 180.0 (C), 159.0 (C), 141.5 (C), 134.4 (CH), 133.4 (CH), 132.0 (C), 131.1 (C), 126.7 (CH), 126.2 (CH), 123.4 (CH), 111.2 (CH), 63.0 (CH_2_), 50.3 (CH_2_), 32.2 (CH_2_), 19.8 (CH_2_), 13.5 (CH_3_); EIMS *m*/*z* (%) 311 ([M^+^], 55); 283 (67); 139 (31); 110 (100); 89 (62); 76 (30); 68 (57); 57 (78); 54 (42); HREIMS: 311.1267 (calcd for C_17_H_17_N_3_O_3_ 311.1270); FT-IR (ATR) ν_max_: 2963, 2934, 2114, 1730, 1686, 1655, 1611, 1465, 1266, 1246, 1046, 1013, 858 cm^−1^.

### 3.4. 2-[(1-Undecyl-triazol-4-yl)methoxy]-naphthalene-1,4-dione (**3b**)

Following the experimental procedure described in method A, from 113.5 mg (0.57 mmol) of 1-azidoundecane (**2b**) and 121.9 mg (0.57 mmol) of the alkyne, we synthesized compound **3b** (208.4 mg, 89%). ^1^H-NMR (CDCl_3_, 400 MHz) δ 7.97 (2H, m), 7.73 (1H, s), 7.63 (2H, m), 6.35 (1H, s), 5.18 (2H, s), 4.29 (2H, t, *J* = 7.2 Hz), 1.84 (2H, t, *J* = 6.4 Hz), 1.25 (2H, brs), 1.17 (14H, brs), 0.80 (3H, m); ^13^C-NMR (CDCl_3_, 100 MHz) δ 184.6 (C), 179.8 (C), 158.8 (C), 141.2 (C), 134.2 (CH), 133.3 (CH), 131.8 (C), 130.9 (C), 126.5 (CH), 126.0 (CH), 123.5 (CH), 111.1 (CH), 62.9 (CH_2_), 50.5 (CH_2_), 31.8 (CH_2_), 30.1 (CH_2_), 29.5 (CH_2_), 29.4 (CH_2_), 29.3 (CH_2_), 28.9 (CH_2_), 27.9 (CH_2_), 26.4 (CH_2_), 22.6 (CH_2_), 14.0 (CH_3_); EIMS *m*/*z* (%) 409 ([M^+^], 41); 254 (12); 236 (34); 208 (100); 173 (30); 105 (19); 89 (13); 82 (10); 70 (18); 57 (33); 55 (25); HREIMS: 409.2356 (calcd for C_24_H_31_N_3_O_3_ 409.2365); FT-IR (ATR) ν_max_: 2921, 2852, 1721, 1679, 1652, 1609, 1464, 1243, 1042, 1009, 832 cm^−1^.

### 3.5. 2-[(1-Bencyl-triazol-4-yl)methoxy]-naphthalene-1,4-dione (**3c**)

Following the experimental procedure described in method A, from 76.5 mg (0.57 mmol) of 1-azidomethylbenzene **2c** and 121.9 mg (0.57 mmol) of the alkyne, we synthesized compound **3c** (169.4 mg, 85%). ^1^H-NMR (CDCl_3_, 400 MHz) δ 8.01 (2H, t, *J* = 7.3 Hz), 7.71 (2H, m), 7.43 (1H, s), 7.37 (3H, m), 7.29 (2H, m), 6.39 (1H, s), 5.54 (2H, s), 5.21 (2H, s); ^13^C-NMR (CDCl_3_, 100 MHz) δ 184.7 (C), 179.8 (C), 158.8 (C), 141.8 (C), 134.3 (CH), 134.3 (C), 133.3 (CH), 131.8 (C), 130.9 (C), 129.1 (2xCH), 128.9 (CH), 128.2 (2 CH), 126.6 (CH), 126.1 (CH), 123.8 (CH), 111.1 (CH), 62.9 (CH_2_), 54.4 (CH_2_); EISM *m*/*z* (%) 345 ([M^+^], 9); 317 (18); 198 (6); 172 (9); 144 (40); 104 (8); 91 (100), 89 (10); 68 (13); 65 (7); HREISM: 345.1120 (calcd for C_20_H_15_N_3_O_3_ 345.1113); FT-IR (ATR) ν_max_: 3052, 1682, 1653, 1616, 1454, 1336, 1307, 1207, 1122, 1047, 890 cm^−1^.

### 3.6. 2-[4-[(1,4-Dioxo-2-naphthyl)oximethyl]triazol-1-yl]-naphthalene-1,4-dione (**3d**)

Following the experimental procedure described in method B, from 114.7 mg (0.57 mmol) of 2-azidonaphthoquinone, 121.9 mg (0.57 mmol) of the alkyne, and 16.4 mg (0.086 mmol) of CuI in 5 mL of dried CH_3_CN, we synthesized compound **3d** (75.4 mg, 32%). ^1^H-NMR (CDCl_3_, 400 MHz) δ 8.88 (1H, s, H-5′), 8.21 (1H, dd, *J* = 8.1, 1.8 Hz), 8.16 (1H, d, *J* = 8.1, 1.8 Hz), 8.10 (2H, m), 7.86 (2H, m), 7.78 (1H, s), 7.74 (2H, m), 6.46 (1H, s), 5.36 (2H, s); ^13^C-NMR (CDCl_3_, 100 MHz) δ 184.8 (C), 183.7 (C), 179.9 (C), 179.3 (C), 158.9 (C), 142.7 (C), 139.2 (C), 135.3 (CH), 134.6 (CH), 134.5 (CH), 133.6 (CH), 132.1 (C), 131.6 (C), 131.2 (C), 131.1 (C), 127.5 (CH), 127.2 (CH), 126.9 (CH), 126.8 (CH), 126.4 (CH), 126.3 (CH), 111.4 (CH), 62.6 (CH_2_, OCH_2_); EIMS *m*/*z* (%) 411 ([M^+^], 29); 382 (11); 329 (52); 327 (63); 299 (26); 254 (46); 239 (30); 209 (100); 173 (58); 156 (50); 128 (32); 115 (14); 105 (45); 89 (40); 76 (45); 54 (7); HREIMS: 411.0851 (calcd for C_23_H_13_N_3_O_5_ 411.0855); FT-IR (ATR) ν_max_: 3188, 3077, 2929, 1681, 1658, 1584, 1337, 1242, 1011, 940, 844 cm^−1^.

### 3.7. 3-Amino-2-[4-[(1,4-dioxo-2-naphtyl)oximethyl]triazol-1-yl]-naphthalene-1,4-dione (**3e**)

Following the experimental procedure described in method A, from 23.6 mg (0.11 mmol) of the azide and 24.0 mg (0.11 mmol) of the alkyne, we synthesized compound **3e** (32.5 mg, 69%). ^1^H-NMR (CDCl_3_, 400 MHz) δ 8.92 (1H, s), 8.22 (12H, d, *J* = 7.8 Hz), 8.12 (3H, m), 7.83 (1H, t, *J* = 7.1 Hz), 7.75 (3H, m), 6.49 (1H, s, H-3), 5.37 (2H, s); ^13^C-NMR (CDCl_3_, 150 MHz) δ 185.0 (C), 180.6 (C), 180.0 (C), 175.9 (C), 159.1 (C), 141.3 (C), 140.3 (C), 135.8 (CH), 134.5 (CH), 133.6 (CH), 133.2 (CH), 132.2 (C), 132.1 (C), 131.2 (C), 129.7 (C), 127.3 (CH), 127.0 (CH), 126.9 (CH), 126.8 (CH), 126.4 (CH), 112.5 (C), 111.4 (CH), 62.8 (CH_2_); EIMS *m*/*z* (%) 426 ([M^+^], 1); 225 (100); 224 (30); 173 (21); 171 (11); 153 (4); 105 (22); 104 (9); 89 (13); 63 (2); HREIMS: 426.0949 (calcd for C_23_H_14_N_4_O_5_ 426.0964); FT-IR (ATR) ν_max_: 3597, 3439, 3288, 3068, 1685, 1610, 1576, 1359, 1309, 1279, 1208, 1043, 1007, 855 cm^−1^.

### 3.8. 2-[[1-[2-Oxo-2-(4-phenylphenyl)ethyl]triazol-4-yl]-methoxy]naphthalene-1,4-dione (**3f**)

Following the experimental procedure described in method B, from 135.1 mg (0.57 mmol) of azide **2f**, 121.9 mg (0.57 mmol) of the alkyne, and 16.4 mg (0.086 mmol) of CuI, we synthesized compound **3f** (32.2 mg, 13%). ^1^H-NMR (CDCl_3_, 400 MHz) δ 8.12 (2H, dd, *J* = 7.3, 0.9 Hz), 8.08 (2H, d, *J* = 8.4 Hz), 7.95 (1H, s), 7.77 (2H, d, *J* = 8.4 Hz), 7.73 (2H, m), 7.64 (2H, d, *J* = 7.3 Hz), 7.50 (2H, m), 7.44 (1H, m), 6.47 (1H, s), 5.91 (2H, s), 5.33 (2H, s); ^13^C-NMR (CDCl_3_, 125 MHz) δ 185.0 (C), 184.9 (C), 179.8 (C), 159.1 (C), 147.7 (C), 145.9 (C), 139.5 (C), 134.4 (CH), 133.5 (CH), 132.6 (C), 132.1 (C), 131.3 (C), 129.2 (2 CH), 128.9 (2 CH), 128.9 (CH), 127.9 (2 CH), 127.5 (2 CH), 126.8 (CH), 126.3 (CH), 125.8 (CH), 111.5 (CH), 111.4 (CH), 62.9 (CH_2_), 56.2 (CH_2_); EIMS *m*/*z* (%) 449 ([M^+^], 2); 198 (6); 182 (14); 181 (100); 153 (14); 152 (19); 105 (6); 89 (2); 77 (3); 76 (3); HREIMS: 449.1391 (calcd for C_27_H_19_N_3_O_4_ 449.1376); FT-IR (ATR) ν_max_: 2924, 2853, 1682, 1652, 1605, 1447, 1355, 1236, 1157, 1014, 936, 844 cm^−1^.

### 3.9. 2-[[1-(2-Oxochromen-4-yl)triazol-4-yl]methoxy]-naphthalene-1,4-dione (**3g**)

Following the experimental procedure described in method B, compound **3g** was synthesized from 48.6 mg (0.26 mmol) of 4-azide-coumarin (**2g**), 48.7 mg (0.26 mmol) of the alkyne, and 16.4 mg (0.086 mmol) of CuI. After 72 h of reaction at room temperature, the obtained solid was filtered and washed with DCM, yielding **3g** (61.7 mg, 59%) without further purification. ^1^H-NMR (DMSO-*d*_6_, 500 MHz) δ 9.02 (1H, s), 8.04 (1H, d, *J* = 7.6 Hz), 8.01 (1H, d, *J* = 7.5 Hz), 7.84 (4H, m), 7.61 (1H, d, *J* = 8.5 Hz), 7.47 (1H, t, *J* = 7.8 Hz), 7.02 (1H, s), 6.71 (1H, s), 5.43 (2H, s); ^13^C-NMR (DMSO-*d*_6_, 150 MHz) δ 185.2 (C), 180.0 (C), 160.1 (C), 159.3 (C), 154.0 (C), 146.4 (C), 142.2 (C), 135.2 (CH), 134.3 (CH), 134.2 (CH), 131.9 (C), 131.2 (C), 127.7 (CH), 126.7 (CH), 126.1 (CH), 125.8 (CH), 125.6 (CH), 117.7 (CH), 114.7 (C), 111.5 (CH), 111.3 (CH), 62.2 (CH_2_); EIMS *m*/*z* (%) 399 ([M^+^], 61); 198 (18); 145 (13); 127 (11); 118 (10); 115 (21); 104 (28); 101 (36); 90 (22); 89 (100); 77 (28); 76 (46); 63 (51); 51 (24); HREIMS: 399.0855 (calcd for C_22_H_13_N_3_O_5_ 399.0855); FT-IR (ATR) ν_max_: 3250, 3057, 2992, 2127, 1715, 1605, 1490, 1385, 1249, 1182, 1016, 937, 852, 749 cm^−1^.

### 3.10. 2-(2-Dimethylaminoethyl)-6-{4-[(1,4-dioxo-2-naphthyl)-oximethyl]triazol-1-yl}benzo[de] isoquinoline-1,3-dione (**3h**)

Following the experimental procedure described in method B, compound **3h** was synthesized from 36.3 mg (0.17 mmol) of azide **2h**, 52.9 mg (0.17 mmol) of the alkyne, and 16.4 mg (0.086 mmol) of CuI in dried THF. The corresponding residue was purified by preparative with DCM/MeOH 10%, yielding **3h** (54.7 mg, 61%), and 2-amino-3-methylnaphthalene-1,4-dione (9.3 mg, 19%). ^1^H-NMR (CDCl_3_, 500 MHz) δ 8.71 (2H, m), 8.25 (1H, s), 8.23 (1H, d, *J* = 8.6 Hz), 8.12 (2H, m), 7.85 (2H, m), 7.76 (1H, t, *J* = 7.4 Hz), 7.74 (1H, t, *J* = 7.4 Hz), 6.51 (1H, s), 5.43 (2H, s), 4.37 (2H, t, *J* = 6.8 Hz), 2.70 (2H, t, *J* = 6.8 Hz), 2.37 (6H, s); ^13^C-NMR (CDCl_3_, 125 MHz) δ 184.8 (C), 179.6 (C), 163.8 (C), 163.2 (C), 159.0 (C), 142.6 (C), 137.9 (C), 134.6 (CH), 133.6 (CH), 132.5 (CH), 132.1 (C), 131.2 (C), 130.8 (CH), 129.3 (CH), 129.3 (C), 128.9 (CH), 126.9 (CH), 126.6 (C), 126.4 (CH), 126.0 (CH), 124.4 (C), 123.8 (CH), 123.3 (C), 111.5 (CH), 62.9 (CH_2_), 57.1 (CH_2_), 45.8 (2 CH_3_), 38.5 (CH_2_); HRMS-ES (+): 544.1587 (calcd for C_29_H_23_N_5_O_5_Na [M + Na]^+^ 544.1597); FT-IR (ATR) ν_max_: 3350, 3200, 2924, 2823, 1657, 1604, 1345, 1239, 1039, 1012, 858 cm^−1^.

### 3.11. 5-[4-[(1,4-Dioxo-2-naphthyl)oximethyl]triazol-1-yl]-benzo[de]isoquinoline-1,3-dione (**3i**)

Following the experimental procedure described in method B, compound **3i** was synthesized from 74.6 mg (0.24 mmol) of azide **2i**, 51.2 mg (0.24 mmol) of the alkyne, and 16.4 mg (0.086 mmol) of CuI in 8 mL of dried THF. After 72 h of reaction at room temperature, the obtained solid was filtered and washed with *n*-hexane, yielding compound **3i** (61.7 mg, 57%) without further purification. ^1^H-NMR (DMSO-*d*_6_, 500 MHz) δ 9.34 (1H, s), 9.05 (1H, s), 8.92 (1H, s), 8.55 (2H, m), 7.99 (3H, m), 7.85 (2H, m), 6.69 (1H, s), 5.42 (2H, s), 4.22 (2H, brs); ^13^C-NMR (DMSO-*d*_6_, 125 MHz) δ 184.5 (C), 179.4 (C), 163.1 (C), 162.7 (C), 158.8 (C), 135.6 (CH), 134.5 (CH), 133.7 (CH), 131.7 (C), 131.5 (CH), 131.4 (C), 128.7 (CH), 126.1 (CH), 125.6 (CH), 124.1 (CH), 124.1 (C), 123.9 (CH), 122.3 (CH), 122.2 (C), 111.0 (CH), 62.2 (OCH_2_) (four quaternary carbons of compound **3i** were not observed in the ^13^C-NMR spectrum due to the fluxional effect); EIMS *m*/*z* (%) 450 ([M^+^], 3); 368 (30); 264 (20); 256 (23); 236 (42); 137 (31); 121 (21); 97 (83); 83 (85); 57 (100); HREIMS: 450.0978 (calcd for C_25_H_14_N_4_O_5_ 450.0954); FT-IR (ATR) ν_max_: 3117, 2980, 2824, 2772, 1705, 1663, 1615, 1525, 1338, 1247, 1214, 1058, 899, 790 cm^−1^.

### 3.12. 2-[(1-Phenyltriazol-4-yl)methoxy]-naphthalene-1,4-dione (**3j**)

Following the general procedure described in method B, 52.7 mg (0.25 mmol) of the alkyne and 25 mol % of CuI were stirred at room temperature for 72 h. Then, the solvent was removed at reduced pressure and the solid was washed with *n*-hexane, yielding triazole **3j** (24.8 mg, 30%) without further purification. ^1^H-NMR (CDCl_3_, 400 MHz) δ 8.10 (1H, s), 8.08 (2H, m), 7.73 (4H, m), 7.54 (2H, m), 7.47 (1H, t, *J* = 7.4 Hz), 6.47 (1H, s), 5.35 (2H, s); ^13^C-NMR (CDCl_3_, 100 MHz) δ 184.8 (C), 180.0 (C), 159.0 (C), 142.4 (C), 136.9 (C), 134.5 (CH), 133.5 (CH), 132.8 (C), 131.2 (C), 130.0 (2 CH), 129.3 (CH), 126.8 (CH), 126.4 (CH), 121.9 (CH), 120.8 (2 CH), 111.4 (CH), 63.0 (CH_2_); EIMS *m*/*z* (%) 331 ([M^+^], 12); 303 (12); 274 (10); 173 (8); 146 (7); 130 (100); 89 (11); 77 (37); 57 (9); HREIMS: 331.0954 (calcd for C_19_H_13_N_3_O_3_ 331.0957); FT-IR (ATR) ν_max_: 3137, 2923, 1679, 1643, 1600, 1504, 1258, 1232, 1038, 1007, 865 cm^−1^.

### 3.13. 2-[(1-(2-Methoxyphenyl)-triazol-4-yl)methoxy]-naphthalene-1,4-dione (**3k**)

Following the general procedure described in method B, 53.9 mg (0.25 mmol) of the alkyne, 37.8 mg (0.25 mmol) of 2-methoxybenzylazide, and 25 mol % of CuI were stirred at room temperature for 72 h. Then, the solvent was removed at reduced pressure and the solid was washed with *n*-hexane, yielding triazole **3k** (80.3 mg, 88%) without further purification. ^1^H-NMR (CDCl_3_, 400 MHz) δ 8.31 (1H, s), 8.16 (1H, dd, *J* = 7.6, 1.4 Hz), 8.10 (1H, dd, *J* = 7.6, 1.4 Hz), 7.73 (1H, dd, *J* = 7.8, 1.5 Hz), 7.73 (2H, m), 7.44 (1H, td, *J* = 7.8, 1.5 Hz), 7.11 (2H, m), 6.50 (1H, s), 5.35 (2H, s), 3.90 (3H, s); ^13^C-NMR (CDCl_3_, 100 MHz) δ 184.9 (C), 180.1 (C), 159.1 (C), 147.1 (C), 134.4 (CH), 133.5 (CH), 132.1 (C), 131.2 (C), 130.5 (CH), 126.8 (CH), 126.3 (CH), 126.0 (CH), 125.6 (CH), 122.3 (C), 121.4 (CH), 112.4 (CH), 111.4 (CH), 63.1 (CH_2_), 56.1 (CH_3_); EIMS *m*/*z* (%) 361 ([M^+^], 5); 303 (4); 174 (10); 160 (100); 145 (15); 92 (9); 89 (10); 77 (18); 64 (5); HREIMS: 361.1060 (calcd for C_20_H_15_N_3_O_4_ 361.1063); FT-IR (ATR) ν_max_: 3173, 2933, 2837, 1682, 1646, 1599, 1508, 1333, 1242, 1005, 878, 722 cm^−1^.

### 3.14. 2-[(1-(3-Methoxyphenyl)-triazol-4-yl)methoxy]-naphthalene-1,4-dione (**3l**)

Following the general procedure described in method B, 52.9 mg (0.25 mmol) of the alkyne, 37.3 mg (0.25 mmol) of 3-methoxybenzylazide, and 25% mol of CuI were stirred at room temperature for 72 h. Then, the solvent was removed at reduced pressure and the solid was washed with *n*-hexane, yielding triazole **3l** (84.6 mg, 94%) without further purification. ^1^H-NMR (CDCl_3_, 500 MHz) δ 8.17 (1H, s), 8.13 (1H, d, *J* = 7.5 Hz), 8.09 (1H, d, *J* = 7.5 Hz), 7.77 (1H, t, *J* = 7.4 Hz), 7.72 (1H, t, *J* = 7.4 Hz), 7.43 (1H, t, *J* = 8.3 Hz), 7.34 (1H, brs), 7.27 (1H, brs), 7.00 (1H, dd, *J* = 8.3, 2.0 Hz), 6.47 (1H, s), 5.34 (2H, s), 3.89 (3H, s); ^13^C-NMR (CDCl_3_, 100 MHz) δ 184.8 (C), 180.2 (C), 160.8 (C), 159.0 (C), 142.3 (C), 137.9 (C), 134.5 (CH), 133.5 (CH), 132.1 (C), 131.2 (C), 130.7 (CH), 126.8 (CH), 126.4 (CH), 122.0 (CH), 115.2 (CH), 112.7 (CH), 111.4 (CH), 106.6 (CH), 63.0 (CH_2_), 55.8 (CH_3_); EIMS *m/z* (%) 361 ([M^+^], 21); 304 (6); 160 (100); 159 (23); 145 (12); 117 (18); 107 (20); 92 (22); 89 (28); 77 (22); 64 (11); HREIMS: 361.1059 (calcd for C_20_H_15_N_3_O_4_ 361.1063); FT-IR (ATR) ν_max_: 3153, 2930, 1680, 1651, 1605, 1497, 1461, 1304, 1236, 1155, 1041, 1007, 941, 841 cm^−1^.

### 3.15. 2-[(1-(3-Trifluoromethyl-phenyl)-triazol-4-yl)methoxy]-naphthalene-1,4-dione (**3m**)

Following the general procedure described in method B, 52.8 mg (0.25 mmol) of the alkyne, 50.5 mg (0.27 mmol) of 3-trifluoromethylbenzylazide, and 25 mol % of CuI were stirred at room temperature for 72 h. Then, the solvent was removed at reduced pressure and the solid was washed with *n*-hexane, yielding triazole **3m** (45.8 mg, 46%) without further purification. ^1^H-NMR (CDCl_3_, 500 MHz) δ 8.24 (1H, s), 8.13 (1H, dd, *J* = 7.5, 0.8 Hz), 8.09 (1H, dd, *J* = 7.5, 0.8 Hz), 8.05 (1H, brs), 7.99 (1H, brd, *J* = 7.9 Hz), 7.74 (4H, m), 6.45 (1H, s), 5.36 (2H, s); ^13^C-NMR (CDCl_3_, 125 MHz) δ 184.8 (C), 180.0 (C), 158.8 (C), 142.9 (C), 137.2 (C), 134.6 (CH), 133.6 (CH), 132.7 (C, *J* = 36.7 Hz), 132.0 (C), 131.1 (C), 130.8 (CH), 126.8 (CH), 126.4 (CH), 125.8 (CH, *J* = 3.6 Hz), 123.8 (CH), 121.9 (CH), 117.7 (CH, *J* = 3.9 Hz), 111.4 (CH), 62.9 (CH_2_); EIMS *m*/*z* (%) 339 ([M^+^], 8); 371 (9); 342 (7); 198 (100); 197 (22); 178 (10); 174 (14); 145 (39); 105 (11); 89 (10); 76 (6); 63 (2); HREIMS: 339.0819 (calcd for C_20_H_12_N_3_O_4_F_3_ 399.0831); FT-IR (ATR) ν_max_: 3155, 3081, 2937, 1676, 1658, 1609, 1463, 1319, 1246, 1177, 1007, 870, 782, 704 cm^−1^.

### 3.16. 2-[(1-(3-Nitrophenyl)-triazol-4-yl)methoxy]-naphthalene-1,4-dione (**3n**)

Following the general procedure described in method B, 56.3 mg (0.27 mmol) of the alkyne, 43.6 mg (0.27 mmol) of 3-nitrobenzylazide, and 25 mol % of CuI were stirred at room temperature for 72 h. Then, the solvent was removed at reduced pressure and the solid was washed with *n*-hexane, yielding triazole **3n** (90.8 mg, 91%) without further purification. ^1^H-NMR (CDCl_3_, 500 MHz) δ 8.64 (1H, t, *J* = 2.1 Hz), 8.35 (1H, ddd, *J* = 8.1, 2.1, 0.9 Hz), 8.32 (1H, s), 8.20 (1H, ddd, *J* = 8.1, 2.1, 0.9 Hz), 8.14 (1H, dd, *J* = 7.6, 1.4 Hz), 8.11 (1H, dd, *J* = 7.6, 1.4 Hz), 7.79 (1H, t, *J* = 8.1 Hz), 7.75 (2H, m), 6.68 (1H, s), 5.40 (2H, s); ^13^C-NMR (CDCl_3_, 125 MHz) δ 184.8 (C), 180.0 (C), 158.9 (C), 149.2 (C), 143.3 (C), 137.6 (C), 134.6 (CH), 133.6 (CH), 132.1 (C), 131.6 (CH), 131.2 (C), 126.9 (CH), 126.4 (CH), 126.2 (CH, C), 123.7 (CH, C), 121.8 (CH), 115.6 (CH), 111.5 (CH), 62.9 (CH); EIMS *m*/*z* (%) 376 ([M^+^], 11); 348 (11); 175 (100); 174 (34); 173 (34); 145 (11); 129 (69); 128 (23); 105 (21); 101 (16); 92 (10); 89 (34); 76 (57); 63 (12); HREIMS: 376.0807 (calcd for C_19_H_12_N_4_O_5_ 376.0808); FT-IR (ATR) ν_max_: 3080, 2959, 2879, 1672, 1609, 1539, 1346, 1243, 1191, 1041, 1013, 867, 778, 737, 670 cm^−1^.

### 3.17. 2-[(1-(4-Methoxyphenyl)-triazol-4-yl)methoxy]-naphthalene-1,4-dione (**3o**)

Following the general procedure described in method B, 44.8 mg (0.21 mmol) of the alkyne, 31.4 mg (0.21 mmol) of 4-methoxybenzylazide, and 25% mol of CuI were stirred at room temperature for 72 h. Then, the solvent was removed at reduced pressure and the solid was washed with *n*-hexane, yielding triazole **3o** (74.7 mg, 98%) without further purification. ^1^H-NMR (CDCl_3_, 500 MHz) δ 8.13 (1H, dd, *J* = 7.5, 1.2 Hz), 8.10 (1H, dd, *J* = 7.5, 1.1 Hz), 8.08 (1H, s), 7.76 (1H, td, *J* = 7.4, 1.3 Hz), 7.72 (1H, td, *J* = 7.4, 1.3 Hz), 7.64 (1H, d, *J* = 9.0 Hz), 7.03 (2H, d, *J* = 9.0 Hz), 6.47 (1H, s), 5.34 (2H, s), 3.88 (3H, s); ^13^C-NMR (CDCl_3_, 125 MHz) δ 184.8 (C), 180.2 (C), 160.3 (C), 159.0 (C), 142.2 (C), 134.5 (CH), 133.5 (CH), 132.1 (C), 131.2 (C), 130.4 (C), 126.8 (CH), 126.4 (CH), 122.5 (2 CH), 122.1 (CH), 115.0 (2 CH), 111.4 (CH), 63.1 (CH_2_), 55.8 (CH_3_); EIMS *m*/*z* (%) 361 ([M^+^], 14); 161 (12); 160 (93); 159 (27); 146 (12); 145 (34); 144 (24); 117 (49); 105 (22); 101 (24); 92 (55); 89 (100); 77 (53); 76 (47); 64 (40); 63 (37); 51 (17); HREIMS: 361.1057 (calcd for C_20_H_15_N_3_O_4_ 361.1063); FT-IR (ATR) ν_max_: 3151, 2937, 2838, 1679, 1655, 1609, 1515, 1308, 1242, 1018, 830, 720 cm^−1^.

### 3.18. 2-[(1-(4-Hydroxymethyl-phenyl)-triazol-4-yl)methoxy]-naphthalene-1,4-dione (**3p**)

Following the general procedure described in method B, 59.8 mg (0.28 mmol) of the alkyne, 42.0 mg (0.28 mmol) of 1-azido-4-(hydroxymethyl)bencene, and 25% mol of CuI were stirred at room temperature for 72 h. Then, the solvent was removed at reduced pressure and the solid was washed with *n*-hexane, yielding triazole **3p** (84.8 mg, 85%) without further purification. ^1^H-NMR (DMSO-*d*_6_, 500 MHz) δ 9.00 (1H, s), 8.02 (1H, d, *J* = 7.5 Hz), 8.00 (1H, d, *J* = 7.5 Hz), 7.86 (4H, m), 7.55 (2H, d, *J* = 8.2 Hz), 6.68 (1H, s), 5.39 (1H, t, *J* = 5.6 Hz), 5.36 (2H, s), 4.59 (2H, d, *J* = 5.6 Hz); ^13^C-NMR (CDCl_3_, 125 MHz) δ 184.5 (C, C-4), 179.4 (C, C-1), 158.8 (C, C-2), 143.5 (C, C-4′), 135.0 (C, C-4′′), 134.5 (CH, C-6), 133.6 (CH, C-7), 131.4 (C, C-4a), 130.8 (C, C-8a), 127.6 (2 CH, C-3′′+C-5′′), 127.6 (C, C-1′′), 126.1 (CH, C-5), 125.5 (CH, C-8), 123.7 (CH, C-5′), 120.0 (2 CH, C-2′′ + C-6′′), 111.0 (CH, C-3), 62.2 (CH_2_, OCH_2_), 62.2 (CH_2_, CH_2_OH); EIMS *m*/*z* (%) 361 ([M^+^], 15); 160 (45); 158 (8); 142 (16); 130 (53); 105 (28); 101 (20); 89 (100); 77 (94); 76 (49); 75 (20); 63 (32); 51 (31); HREIMS: 361.1057 (calcd for C_20_H_15_N_3_O_4_ 361.1063); FT-IR (ATR) ν_max_: 3475, 3106, 2932, 2878, 1684, 1659, 1610, 1522, 1246, 1207, 1049, 820, 725 cm^−1^.

### 3.19. 2-[(1-(4-Mercaptophenil)-triazol-4-yl)methoxy]-naphthalene-1,4-dione (**3q**)

Following the general procedure described in method B, 47.5 mg (0.22 mmol) of the alkyne, 33.8 mg (0.22 mmol) of 4-mercaptobenzylazide, and 25% mol of CuI were stirred at room temperature for 72 h. Then, the solvent was removed at reduced pressure and the solid was washed with *n*-hexane, yielding triazole **3q** (71.5 mg, 88%) without further purification. ^1^H-NMR (DMSO-*d*_6_, 500 MHz) δ 9.01 (1H, s), 8.01 (2H, m), 7.67 (2H, d, *J* = 8.4 Hz), 7.86 (2H, m), 7.80 (2H, d, *J* = 8.4 Hz), 6.66 (1H, s), 5.35 (2H, s); ^13^C-NMR (DMSO-*d*_6_, 125 MHz) δ 184.5 (C, C-4), 179.9 (C, C-1), 159.2 (C, C-2), 142.1 (C, C-4′), 136.4 (C, C-1′′), 134.5 (CH, C-6), 133.6 (CH, C-7), 131.5 (C, C-4a), 131.4 (C, C-4′′), 130.8 (C, C-8a), 128.7 (2 CH, C-3′′ + C-5′′), 126.1 (CH, C-5), 125.5 (CH, C-8), 123.7 (CH, C-5′), 121.4 (2 CH, C-2′′ + C-6′′), 111.0 (CH, C-3), 62.1 (CH_2_, OCH_2_); HRMS-ES (−): 362.0598 (calcd for C_19_H_12_N_3_O_3_S [M − H^+^]^−^ 362.0599); FT-IR (ATR) ν_max_: 3294, 3068, 2931, 2128, 1651, 1607, 1496, 1330, 1240, 1012, 824, 720 cm^−1^.

### 3.20. 2-[(1-(4-Fluoromethyl-phenyl)-triazol-4-yl)methoxy]-naphthalene-1,4-dione (**3r**)

Following the general procedure described in method B, 46.5 mg (0.22 mmol) of the alkyne, 34.3 mg (0.22 mmol) of 1-azido-4-fluorobenzene, and 25% mol of CuI were stirred at room temperature for 72 h. Then, the solvent was removed at reduced pressure and the solid was washed with *n*-hexane, yielding triazole **3r** (77.6 mg, 78%) without further purification. ^1^H-NMR (CDCl_3_, 500 MHz) δ 8.14 (1H, m), 8.13 (1H, s), 8.10 (1H, d, *J* = 7.6 Hz), 7.72 (4H, m), 7.23 (2H, t, *J* = 8.6 Hz), 6.46 (1H, s), 5.34 (2H, s); ^13^C-NMR (CDCl_3_, 125 MHz) δ 184.8 (C), 180.0 (C), 162.8 (C-F, *J* = 245.4 Hz), 159.0 (C), 142.6 (C), 134.5 (CH), 133.6 (CH), 132.1 (C), 131.2 (C), 126.9 (CH), 126.4 (CH), 122.9 (2 CH, *J* = 8.4 Hz), 122.1 (CH), 117.0 (2 CH, *J* = 23.0 Hz), 111.4 (CH), 63.0 (C); EIMS *m*/*z* (%) 349 ([M^+^], 8); 173 (8); 148 (100); 129 (9); 122 (14); 105 (13); 101 (33); 95 (71); 89 (66); 76 (31); 75 (45); 63 (14); 51 (19); HREIMS: 349.0848 (calcd for C_19_H_12_N_3_O_3_F, [M^+^] 349.0863); FT-IR (ATR) ν_max_: 3226, 3060, 2933, 2874, 2129, 1683, 1643, 1607, 1331, 1241, 1118, 1015, 940, 723 cm^−1^.

### 3.21. 2-[(1-(3-Fluoro-4-methoxyphenyl)-triazol-4-yl)methoxy]-naphthalene-1,4-dione (**3s**)

Following the general procedure described in method B, 42.8 mg (0.21 mmol) of the alkyne, 42.8 mg (0.21 mmol) of 3-fluoro-4-methoxybenzylazide, and 25% mol of CuI were stirred at room temperature for 72 h. Then, the solvent was removed at reduced pressure and the solid was washed with *n*-hexane, yielding triazole **3s** (89.3 mg, 89%) without further purification. ^1^H-NMR (CDCl_3_, 400 MHz) δ 8.13 (1H, dd, *J* = 7.3, 1.4 Hz), 8.10 (1H, dd, *J* = 7.3, 1.4 Hz), 8.09 (1H, s), 7.75 (1H, td, *J* = 7.3, 1.4 Hz), 7.73 (1H, td, *J* = 7.3, 1.4 Hz), 7.54 (1H, dd, *J* = 11.2, 2.5 Hz), 7.45 (1H, brd, *J* = 8.7 Hz), 7.09 (1H, t, *J* = 8.7 Hz), 6.45 (1H, s), 5.33 (2H, s), 3.96 (3H, s); ^13^C-NMR (CDCl_3_, 100 MHz) δ 184.8 (C), 180.0 (C), 159.0 (C), 152.5 (C-F, *J* = 248.0 Hz), 148.4 (C, *J* = 10.3 Hz), 142.4 (C), 134.5 (CH), 133.6 (CH), 132.1 (C), 131.2 (C), 130.0 (C, *J* = 34.9 Hz), 126.8 (CH), 126.4 (CH), 121.9 (CH), 126.7 (CH, *J* = 13.4 Hz), 113.9 (CH), 111.4 (CH), 110.0 (CH, *J* = 90.6 Hz), 63.0 (CH_2_), 56.7 (CH_3_); EIMS *m*/*z* (%) 379 ([M^+^], 3); 322 (4); 178 (100); 177 (19); 163 (13); 135 (5); 110 (6); 89 (9); 82 (9); 82 (7); 77 (6); 76 (6); 63 (3); HREIMS: 379.00970 (calcd for C_20_H_14_N_3_O_4_F 379.0968); FT-IR (ATR) ν_max_: 3078, 2322, 2289, 2046, 1681, 1652, 1608, 1521, 1451, 1242, 1202, 1047, 1013, 859, 719 cm^−1^.

### 3.22. 2-[(1-(1H-Indol-5-yl)-triazol-4-yl)methoxy]-naphthalene-1,4-dione (**3t**)

Following the general procedure described in method B, 24.8 mg (0.16 mmol) of the alkyne, 33.3 mg (0.16 mmol) of 5-azido-1*H*-indole, and 25 mol % of CuI were stirred at room temperature for 72 h. Then, the solvent was removed at reduced pressure and the solid was washed with *n*-hexane, yielding triazole **3t** (48.8 mg, 84%) without further purification. ^1^H-NMR (DMSO-*d*_6_, 500 MHz) δ 11.46 (1H, brs), 8.92 (1H, s), 8.04 (1H, s), 8.01 (2H, m), 7.86 (2H, m), 7.59 (2H, brs), 7.52 (1H, s), 6.69 (1H, s), 6.59 (1H, s), 5.35 (2H, s); ^13^C-NMR (DMSO-*d*_6_, 125 MHz) δ 184.5 (C), 179.5 (C), 158.9 (C), 141.5 (C), 135.6 (C), 134.5 (CH), 133.6 (CH), 131.5 (C), 130.8 (C), 129.3 (C), 127.7 (CH), 127.6 (C), 126.1 (CH), 125.5 (CH), 124.0 (CH), 114.4 (CH), 112.3 (2 CH), 111.0 (CH), 101.9 (CH), 62.4 (CH_2_); EIMS *m*/*z* (%) 370 ([M^+^], 6); 174 (32); 169 (100); 168 (62); 142 (21); 116 (80); 105 (47); 89 (61); 77 (21); 76 (26); 63 (16); 51 (11); HREIMS: 370.1055 (calcd for C_21_H_14_N_4_O_3_, 370.1066); FT-IR (ATR) ν_max_: 3342, 3163, 1683, 1647, 1606, 1349, 1239, 1208, 1057, 1014, 866, 727 cm^−1^.

### 3.23. 2-{[1-(E)-2-(4-Trifluorometyl-phenyl)vinyl)-triazol-4-yl]methoxy}-naphthalene-1,4-dione (**3u**)

Following the general procedure described in method B, 66.6 mg (0.31 mmol) of the alkyne, 66.9 mg (0.31 mmol) of azide **2u**, and 25 mol % of CuI were stirred at room temperature for 72 h. Then, the solvent was removed at reduced pressure and the solid was washed with *n*-hexane, yielding triazole **3u** (128.7 mg, 96%) without further purification. ^1^H-NMR (DMSO-*d*_6_, 500 MHz) δ 8.78 (1H, s), 8.39 (1H, d, *J* = 14.7 Hz), 8.02 (1H, d, *J* = 7.5 Hz), 7.99 (1H, d, *J* = 7.5 Hz), 7.83 (6H, m), 7.57 (1H, d, *J* = 14.7 Hz), 6.65 (1H, s), 5.36 (2H, s); ^13^C-NMR (DMSO-*d*_6_, 125 MHz) δ 184.5 (C), 179.4 (C), 158.8 (C), 141.7 (C), 138.1 (C), 134.5 (CH), 133.7 (CH), 131.5 (C), 130.8 (C), 128.4 (C), 127.5 (2 CH), 126.1 (CH), 125.7 (2 CH, *J* = 3.4 Hz), 125.5 (CH), 123.4 (CH), 120.0 (CH), 111.0 (CH), 62.1 (CH_2_); HRMS-ES (+): 448.0873 (calcd for C_22_H_14_F_3_N_3_O_3_Na [M + Na]^+^ 448.0874); FT-IR (ATR) ν_max_: 3566, 3066, 1685, 1650, 1608, 1418, 1323, 1244, 1205, 1113, 1016, 931, 814, 723 cm^−1^.

### 3.24. 2-[(4-Butyl)-triazol-1-yl]-naphthalene-1,4-dione (**4a**)

Following the general procedure described in method B, 77.71 µL (0.68 mmol) of 1-hexyne, 67.3 mg (0.34 mmol) of 2-azide-1,4-naphthoquinone, and 20 mol % of CuI were stirred at room temperature for 17 h. Then, the solvent was removed at reduced pressure and the residue was purified by TLC-preparative with *n*-hexane/EtOAc (40%), yielding triazole **4a** (73.5 mg, 77%). ^1^H-NMR (CDCl_3_, 500 MHz) δ 8.38 (1H, s), 8.21 (1H, m), 8.16 (1H, m), 7.84 (2H, m), 7.75 (1H, s), 2.83 (2H, t, *J* = 7.7 Hz), 1.74 (2H, m), 1.44 (2H, m), 0.97 (3H, t, *J* = 7.4 Hz); ^13^C-NMR (CDCl_3_, 125 MHz) δ 184.0 (C), 179.7 (C), 149.7 (C), 139.6 (C), 135.0 (CH), 134.4 (CH), 131.6 (C), 131.3 (C), 127.3 (CH), 126.6 (CH), 126.2 (CH), 122.9 (CH), 31.3 (CH_2_), 25.4 (CH_2_), 22.4 (CH_2_), 13.9 (CH_3_); HRMS-ES (+) 304.1062 (calcd for C_16_H_15_N_3_O_2_Na [M + Na]^+^ 304.1062); FT-IR (ATR) ν_max_: 3164, 3072, 2957, 2928, 1652, 1593, 1291, 1251, 1043, 1016, 985, 829, 784, 715 cm^−1^.

### 3.25. 2-[(4-Phenyl)-triazol-1-yl]-naphthalene-1,4-dione (**4j**)

Following the general procedure described in method B, 47.9 mg (0.24 mmol) of 2-azide-1,4-naphthoquinone, 52.8 µL of phenylacetylene, and 20 mol % of CuI were stirred at room temperature for 48 h. Then, the solvent was removed at reduced pressure and the residue was purified by TLC-preparative with *n*-hexane/EtOAc (40%), yielding triazole **4j** (51.7 mg, 71%). ^1^H-NMR (CDCl_3_, 400 MHz) δ 8.93 (1H, s), 8.36 (1H, d, *J* = 6.6 Hz), 8.19 (1H, d, *J* = 6.6 Hz), 7.95 (2H, d, *J* = 7.4 Hz), 7.87 (2H, m), 7.85 (1H, s), 7.49 (2H, t, *J* = 7.3 Hz), 7.40 (1H, t, *J* = 7.3 Hz); ^13^C-NMR (CDCl_3_, 100 MHz) δ 183.9 (C), 179.6 (C), 149.0 (C), 139.4 (C), 135.2 (CH), 134.5 (CH), 131.7 (C), 131.2 (C), 129.8 (C), 129.1 (2 CH), 128.9 (CH), 127.4 (CH), 126.7 (CH), 126.4 (CH), 126.1 (2 CH), 121.5 (CH); EIMS *m*/*z* (%) 301 ([M]^+^, 2); 274 (20); 273 (100); 217 (10); 157 (13); 129 (19); 101 (24); 89 (5); 76 (9); HREIMS: 301.0850 (calcd for C_18_H_11_N_3_O_2_ 301.0851); FT-IR (ATR) ν_max_: 3152, 2924, 2853, 2194, 1672, 1592, 1415, 1260, 1005, 915, 762, 691 cm^−1^.

### 3.26. 2-[(1-Undecyl-triazol-4-yl)methylamino]-naphthalene-1,4-dione (**7b**)

Following the experimental procedure described in method C, from 56.0 mg (0.28 mmol) of 1-azido-undecane and 28 mg (0.14 mmol) of *N*-propargylated quinone (**6**), we obtained 46.3 mg (80%) of compound **7b**. ^1^H-NMR (CDCl_3_, 500 MHz) δ 8.09 (1H, d, *J =* 7.6 Hz), 8.05 (1H, d, *J =* 7.6 Hz), 7.73 (1H, t, *J =* 7.6 Hz), 7.63 (1H, t, *J =* 7.5 Hz), 7.52 (1H, s), 6.34 (1H, s), 5.82 (1H, s), 4.50 (2H, d, *J =* 5.6 Hz), 4.35 (2H, t, *J =* 7.3 Hz), 1.90 (2H, m), 1.62 (2H, s), 1.27 (14H, m), 0.87 (3H, t, *J =* 7.0 Hz); ^13^C-NMR (CDCl_3_, 125 MHz) δ 183.0 (C), 181.5 (C), 147.5 (C), 134.7 (CH), 133.5 (C), 132.2 (C), 132.1 (CH), 130.5 (C), 121.7 (CH), 126.3 (CH), 126.2 (CH), 101.7 (CH), 50.5 (CH_2_), 38.3 (CH_2_), 31.9 (CH_2_), 30.2 (CH_2_), 29.6 (CH_2_), 29.5 (CH_2_), 29.3 (CH_2_), 29.2 (CH_2_), 28.9 (CH_2_), 26.5 (CH_2_), 22.6 (CH_2_), 14.0 (CH_3_); HRMS-ES (+): 431.2423 (calcd for C_24_H_32_N_4_O_2_Na, [M + Na]^+^ 431.2417); FT-IR (ATR) ν_max_: 3346, 3135, 3079, 2958, 2922, 2853, 1683, 1596, 1570, 1501, 1471, 1442, 1360, 1348, 1316, 1257, 1223, 1153, 1129, 1102, 1079, 1058, 1032, 988, 935, 897, 829 cm^−1^.

### 3.27. 2-[(1-Benzyl-triazol-4-yl)methylamino]-naphthalene-1,4-dione (**7c**)

Following the experimental procedure described in method C, from 40.8 mg (0.28 mmol) of benzylazide and 28 mg (0.14 mmol) of *N*-propargylated naphthoquinone (**6**), we obtained 27.3 mg (86%) of compound **7c**. ^1^H-NMR (CDCl_3_, 500 MHz) δ 8.06 (1H, d, *J =* 7.6 Hz), 8.01 (1H, d, *J =* 7.6 Hz), 7.71 (1H, t, *J =* 7.6 Hz), 7.60 (1H, t, *J =* 7.5 Hz), 7.48 (1H, s), 7.36 (3H, m), 7.27 (2H, m), 6.39 (1H, s), 5.78 (1H, s), 5.53 (2H, s), 4.47 (2H, d, *J =* 5.7 Hz); ^13^C-NMR (CDCl_3_, 125 MHz) δ 182.9 (C), 181.5 (C), 147.5 (C), 143.1 (C), 134.6 (CH), 134.3 (C), 133.4 (C), 132.0 (CH), 130.4 (C), 129.1 (2 CH), 128.8 (CH), 128.1 (2 CH), 126.2 (CH), 126.1 (CH), 121.8 (CH), 101.6 (CH), 54.3 (CH_2_), 38.2 (CH_2_); EIMS *m*/*z* (%) 344 ([M^+^], 43); 225 (78); 173 (37); 146 (16); 105 (24); 104 (16); 91 (100); HREIMS: 344.1261 (calcd for C_20_H_16_N_4_O_2_ 344.1273); FT-IR (ATR) ν_max_: 3330, 3117, 3064, 2921, 2853, 1681, 1592, 1561, 1496, 1455, 1438, 1352, 1311, 1252, 1217, 1152, 1124, 1096, 1075, 1051, 1027, 981, 932, 862, 845, 822 cm^−1^.

### 3.28. 2-[[1-(1,4-Dioxo-2-naphthyl)-triazol-4-yl]methylamino]-naphthalene-1,4-dione (**7d**)

Following the experimental procedure described in method B, from 63.7 mg (0.32 mmol) of 2-azidonaphthoquinone and 66.8 mg (0.32 mmol) of *N*-propargylated naphthoquinone (**6**), we obtained 42.0 mg (32%) of compound **7d**. ^1^H-NMR (CDCl_3_, 400 MHz) δ 8.67 (1H, s), 8.21 (1H, dd, *J* = 8.8, 2.2 Hz), 8.17 (1H, dd, *J* = 8.8, 2.2 Hz), 8.09 (2H, m), 7.84 (2H, m), 7.78 (1H, s), 7.74 (1H, td, *J* = 7.5, 1.1 Hz), 7.64 (1H, t, *J* = 7.5, 1.1 Hz), 6.38 (1H, brs, NH), 5.88 (1H, s), 4.63 (2H, d, *J* = 5.8 Hz); ^13^C-NMR (DMSO-*d*_6_, 150 MHz) δ 184.5 (C), 182.1 (C), 182.0 (C), 179.2 (C), 148.7 (C), 144.6 (C), 140.7 (C), 135.3 (CH), 135.2 (C), 135.0 (C), 133.4 (C), 132.8 (CH), 131.8 (C), 131.7 (C), 130.9 (CH), 127.2 (CH), 126.7 (CH), 126.4 (C), 126.3 (CH), 126.0 (CH), 125.8 (CH), 101.1 (CH), 37.4 (CH_2_); EIMS *m*/*z* (%): 410 ([M^+^], 1); 255 (19); 254 (100); 226 (11); 197 (8); 186 (15); 173 (22); 158 (21); 146 (16); 102 (16); 83 (14); 76 (17); HREIMS: 410.1218 (calcd for C_23_H_14_N_4_O_4_ 410.1015); FT-IR (ATR) ν_max_: 3338, 3152, 3072, 2932, 2857, 1661, 1604, 1513, 1352, 1294, 1186, 1038, 968, 920, 846 cm^−1^.

### 3.29. 2-(2-Dimethylaminoethyl)-6-{4-[(1,4-dioxo-2-naphthyl)-aminomethyl]triazol-1-yl}benzo[de] isoquinoline-1,3-dione (**7h**)

Following the experimental procedure described in method B, from 31.7 mg (0.1 mmol) of azide **2h** and 18.0 mg (0.09 mmol) of *N*-propargylated naphthoquinone (**6**), we obtained 29.4 mg (66%) of compound **7h**. ^1^H-NMR (CDCl_3_, 500 MHz) δ 8.63 (2H, d, *J =* 7.2 Hz), 8.13 (1H, d, *J =* 8.5 Hz), 8.05 (3H, m), 7.77 (2H, d, *J =* 7.5 Hz), 7.71 (1H, t, *J =* 7.3 Hz), 7.61 (1H, t, *J =* 7.3 Hz), 6.62 (1H, s), 4.69 (2H, d, *J =* 5.7 Hz), 4.34 (2H, t, *J =* 6.7 Hz), 2.71 (2H, t, *J =* 6.5 Hz), 2.37 (6H, s); ^13^C-NMR (CDCl_3_, 125 MHz) δ 183.0 (C), 181.5 (C), 163.5 (C), 163.0 (C), 147.5 (C), 143.6 (C), 137.8 (C), 134.8 (CH), 133.3 (C), 132.2 (CH), 130.5 (CH), 130.4 (C), 129.1 (CH), 129.0 (C), 128.7 (2 CH), 126.4 (C), 126.3 (CH), 126.2 (CH), 124.4 (CH), 124.1 (C), 123.6 (CH), 123.0 (C), 102.0 (CH), 56.9 (CH_2_), 45.6 (2 CH_3_), 38.3 (CH_2_), 38.1 (CH_2_); EIMS *m*/*z* (%): 446 (46); 441 (27); 173 (74); 158 (34); 149 (56); 146 (27); 113 (22); 111 (22); 105 (32); 99 (25); 97 (22); 85 (45); 71 (82); 58 (39); 57 (100); 55 (35); HREIMS: 521.1937 (calcd for C_29_H_24_N_6_O_4_), [M^+^] 521.1937); FT-IR (ATR) ν_max_: 3338, 2967, 2864, 2824, 2259, 1699, 1656, 1599, 1569, 1508, 1477, 1434, 1347, 1305, 1240, 1158, 1121, 1024, 1000, 860, 832 cm^−1^.

### 3.30. 2-[(1-Phenyl-triazol-4-yl)methylamino]-naphthalene-1,4-dione (**7j**)

Following the experimental procedure described in method C, from 31.2 mg (0.24 mmol) of phenylboronic acid and 30.0 mg (0.14 mmol) of *N*-propargylated naphthoquinone (**6**), we obtained 10.9 mg (24%) of compound **7j**. ^1^H-NMR (CDCl_3_, 500 MHz) δ 8.10 (1H, dd, *J =* 7.8, 1.1 Hz), 8.06 (1H, dd, *J =* 7.6, 1.1 Hz), 7.98 (1H, s), 7.73 (3H, m), 7.63 (1H, td, *J =* 7.5, 1.2 Hz), 7.53 (2H, t, *J =* 7.8 Hz), 7.46 (1H, t, *J =* 7.4 Hz), 6.42 (1H, s), 5.88 (1H, s), 4.60 (2H, d, *J =* 5.8 Hz) ppm; ^13^C-NMR (CDCl_3_, 125 MHz) δ 183.1 (C), 181.6 (C), 147.5 (C), 143.5 (C), 136.87 (C), 134.8 (CH), 133.5 (C), 132.2 (CH), 130.5 (C), 129.9 (2 CH), 129.1 (CH), 126.4 (CH), 126.3 (CH), 120.7 (2 CH), 120.2 (CH), 101.9 (CH), 38.2 (CH_2_) ppm; EIMS *m*/*z* (%): 330 ([M^+^], 21), 302 (27), 301 (16), 245 (11), 173 (14), 130 (100), 129 (13), 105 (13), 104 (17), 77 (70), 76 (14), 51 (19); HREIMS: 330.1117 (calcd for C_19_H_14_N_4_O_2_ 330.1117); FT-IR (ATR) ν_max_: 3346, 3132, 3058, 2931, 1671, 1596, 1562, 1501, 1442, 1348, 1306, 1281, 1253, 1222, 1175, 1156, 1122, 1091, 1050, 1024, 983, 927, 856 cm^−1^.

### 3.31. 2-[(1-(2-Methoxyphenyl)-triazol-4-yl)methylamino]-naphthalene-1,4-dione (**7k**)

Following the experimental procedure described in method C, from 38.5 mg (0.24 mmol) of 2-methoxyphenylboronic acid and 30.0 mg (0.14 mmol) of *N*-propargylated naphthoquinone (**6**), we obtained 6.8 mg (14%) of compound **7k**. ^1^H-NMR (CDCl_3_, 500 MHz) δ 8.11 (1H, s), 8.10 (1H, dd, *J =* 7.8, 1.0 Hz), 8.06 (1H, dd, *J =* 7.8, 0.9 Hz), 7.77 (1H, dd, *J =* 7.9, 1.6 Hz), 7.73 (1H, td, *J =* 7.7, 1.1 Hz), 7.63 (1H, t, *J =* 7.54 Hz), 7.43 (1H, td, *J =* 7.8, 1.5 Hz), 7.11 (2H, m), 6.40 (1H, s), 5.89 (1H, s), 4.59 (2H, d, *J =* 5.7 Hz), 3.90 (3H, s) ppm; ^13^C-NMR (CDCl_3_, 125 MHz) δ 183.1(C), 181.6 (C), 151.1 (C), 147.6 (C), 141.9 (C), 134.7 (CH), 133.5 (C), 132.1 (CH), 130.6 (C), 130.3 (CH), 126.3 (CH), 126.2 (CH), 126.1 (C), 125.5 (CH), 124.2 (CH), 121.3 (CH), 112.3 (CH), 101.8 (CH), 56.0 (CH_3_), 38.3 (CH_2_) ppm; EIMS *m*/*z* (%): 360 ([M^+^], 30), 332 (18), 161 (17), 160 (100), 145 (17), 130 (15), 120 (15), 92 (16), 77 (31); HREIMS: 360.1236 (calcd for C_20_H_16_N_4_O_3_ 360.1222); FT-IR (ATR) ν_max_: 3403, 3358, 3139, 3085, 3064, 3011, 2955, 2916, 2848, 1671, 1599, 1565, 1502, 1460, 1355, 1336, 1304, 1283, 1247, 1172, 1156, 1117, 1092, 1074, 1053, 993, 928, 860, 847, 829 cm^−1^.

### 3.32. 2-[(1-(3-Methoxyphenyl)-triazol-4-yl)methylamino]-naphthalene-1,4-dione (**7l**)

Following the experimental procedure described in method C, from 37.2 mg (0.24 mmol) of 3-methoxyphenylboronic acid and 30.0 mg (0.14 mmol) of *N*-propargylated naphthoquinone (**6**), we obtained 27.3 mg (53%) of compound **7l**. ^1^H-NMR (CDCl_3_, 500 MHz) δ 8.08 (1H, d, *J =* 7.3 Hz), 8.04 (1H, d, *J =* 7.2 Hz), 7.97 (1H, s), 7.72 (1H, td, *J =* 7.6, 1.2 Hz), 7.62 (1H, td, *J =* 7.5, 1.2 Hz), 7.40 (1H, t, *J =* 8.17 Hz), 7.32 (1H, t, *J =* 2.1 Hz), 7.23 (1H, dd, *J =* 7.9, 1.2 Hz), 6.97 (1H, dd, *J =* 8.3, 1.9 Hz), 6.43 (1H, s), 5.87 (1H, s), 4.59 (2H, d, *J =* 5.8 Hz), 3.87 (3H, s) ppm; ^13^C-NMR (CDCl_3_, 125 MHz) δ 183.0 (C), 181.5 (C), 160.6 (C), 147.5 (C), 143.4 (C), 137.8 (C), 134.8 (CH), 133.4 (C), 132.2 (CH), 130.6 (CH), 130.5 (C), 126.3 (CH), 126.2 (CH), 120.2 (CH), 114.9 (CH), 112.4 (CH), 106.4 (CH), 101.8 (CH), 55.6 (CH_3_), 38.1 (CH_2_) ppm; EIMS *m*/*z* (%): 360 ([M^+^], 13), 202 (67), 173 (55), 160 (100), 158 (39), 146 (29), 130 (21), 123 (28), 107 (31), 105 (37), 104 (59), 102 (27), 92 (40), 77 (58), 76 (54), 64 (22), 51 (20); HREIMS: 360.1210 (calcd for C_20_H_16_N_4_O_3_ 360.1222); FT-IR (ATR) ν_max_: 3198, 3142, 3091, 3018, 2959, 2922, 2853, 2186, 1695, 1599, 1564, 1489, 1440, 1357, 1335, 1313, 1257, 1168, 1123, 1103, 1046, 1026, 1010, 984, 934, 856, 830 cm^−1^.

### 3.33. 2-[(1-(3-Trifluoromethyl)-triazol-4-yl)methylamino]-naphthalene-1,4-dione (**7m**)

Following the experimental procedure described in method C, from 45.7 mg (0.24 mmol) of 3-(trifluoromethyl)phenylboronic acid and 30.0 mg (0.14 mmol) of *N*-propargylated naphthoquinone (**6**), we obtained 23.7 mg (44%) of compound **7m**. ^1^H-NMR (CDCl_3_, 500 MHz) δ 8.07 (3H, m), 8.01 (1H, s), 7.96 (1H, d, *J =* 7.7 Hz), 7.71 (3H, m), 7.63 (1H, t, *J =* 7.4 Hz), 6.43 (1H, s), 5.87 (1H, s), 4.63 (2H, d, *J =* 5.7 Hz) ppm; ^13^C-NMR (CDCl_3_, 125 MHz) δ 183.1 (C), 181.5 (C), 147.5 (C), 144.1 (C), 137.2 (C), 134.8 (CH), 133.4 (C), 132.6 (C, *J^2^*_C-F_ *=* 33.7 Hz), 132.2 (CH), 130.6 (CH), 130.5 (C), 126.37 (CH), 126.3 (CH), 125.6 (CH, *J^3^*_C-F_ *=* 2.13 Hz), 123.7 (CH), 123.2 (C, *J^1^*_C-F_ = 274 Hz), 120.0 (CH), 117.6 (CH, *J^3^*_C-F_ *=* 3.7 Hz), 102.0 (CH), 38.2 (CH_2_) ppm; EIMS *m*/*z* (%): 398 ([M^+^] 9); 370 (30); 313 (19); 240 (37); 199 (31); 198 (100); 158 (58); 130 (21); 105 (29); 104 (25); 102 (24); 77 (19); 76 (34); HREIMS: 398.1025 (calcd for C_20_H_13_F_3_N_4_O_2_ 398.0991); FT-IR (ATR) ν_max_: 3352, 3130, 3083, 1679, 1601, 1568, 1504, 1458, 1347, 1310, 1286, 1243, 1171, 1117, 1094, 107, 1051, 1001, 976, 901, 878, 811 cm^−1^.

### 3.34. 2-[(1-(3-Nitrophenyl)-triazol-4-yl)methylamino]-naphthalene-1,4-dione (**7n**)

Following the experimental procedure described in method C, from 40.2 mg (0.24 mmol) of 3-nitrophenylboronic acid and 30.0 mg (0.14 mmol) of *N*-propargylated naphthoquinone (**6**), we obtained 14.2 mg (44%) of compound **7n**. ^1^H-NMR (DMSO-*d*_6_, 500 MHz) δ 8.99 (1H, s), 8.70 (1H, s), 8.39 (1H, d, *J =* 8.2 Hz), 8.31 (1H, d, *J =* 8.2 Hz), 8.07 (1H, t, *J =* 6.1 Hz), 8.01 (1H, d, *J =* 7.6 Hz), 7.93 (1H, d, *J =* 7.6 Hz), 7.87 (1H, t, *J =* 8.2 Hz), 7.82 (1H, t, *J =* 7.5 Hz), 7.74 (1H, t, *J =* 7.5 Hz), 5.77 (1H, s), 4.61 (2H, d, *J =* 6.17 Hz) ppm; ^13^C-NMR (DMSO-*d*_6_, 125 MHz) δ 181.6 (C), 181.4 (C), 148.5 (C), 148.3 (C), 144.9 (C), 137.2 (C), 134.8 (CH), 133.0 (C), 132.3 (CH), 131.5 (CH), 130.4 (C), 126.0 (CH), 125.7 (CH), 125.3 (CH), 123.0 (CH), 121.8 (CH), 114.7 (CH), 100.7 (CH), 37.4 (CH_2_) ppm; EIMS *m*/*z* (%): 375 ([M^+^], 39); 348 (23); 347 (61); 346 (34); 300 (28); 198 (23); 182 (28); 175 (72); 174 (23); 173 (64); 146 (35); 145 (34); 130 (26); 129 (100); 128 (25); 105 (58); 104 (33); 102 (41); 101 (32); 92 (21); 89 (31); 77 (37); 76 (79); 75 (26); HREIMS: 375.0983 (calcd for C_19_H_13_N_5_O_4_ 375.0968); FT-IR (ATR) ν_max_: 3360, 3248, 3122, 3083, 3059, 2918, 2850, 1677, 1605, 1567, 1524, 1346, 1304, 1255, 1180, 1159, 1122, 1093, 1075, 1049, 982, 931, 897, 869, 834, 814 cm^−1^.

### 3.35. 2-[(1-(4-Methoxyphenyl)-triazol-4-yl)methylamino]-naphthalene-1,4-dione (**7o**)

Following the experimental procedure described in method C, from 38.5 mg (0.24 mmol) of 3-methoxyphenylboronic acid and 30.0 mg (0.14 mmol) of *N*-propargylated naphthoquinone (**6**), we obtained 29.4 mg (57%) of compound **7o**. ^1^H-NMR (CDCl_3_, 500 MHz) δ 8.08 (1H, dd, *J =* 7.7, 0.9 Hz), 8.04 (1H, dd, *J =* 7.7, 1.1 Hz), 7.90 (1H, s), 7.72 (1H, td, *J =* 7.6, 1.3 Hz), 7.62 (3H, m), 7.00 (2H, m), 6.43 (1H, s), 5.87 (1H, s), 4.58 (2H, d, *J =* 5.8 Hz), 3.86 (3H, s) ppm; ^13^C-NMR (CDCl_3_, 125 MHz) δ 183.0 (C), 181.5 (C), 160.0 (C), 147.5 (C), 143.2 (C), 134.7 (CH), 133.4 (C), 132.1 (CH), 130.6 (C), 130.3 (C), 126.3 (CH), 126.2 (CH), 122.3 (2 CH), 120.3 (CH), 114.9 (2xCH), 101.8 (CH), 55.6 (CH_3_), 38.2 (CH_2_) ppm; EIMS *m*/*z* (%): 360 ([M^+^], 28); 360 (28); 332 (19); 202 (50); 187 (31); 173 (33); 160 (100); 158 (20); 123 (20); 108 (24); 105 (18); 104 (23); 77 (19); HREIMS: 360.1237 (calcd for C_20_H_16_N_4_O_3_ 360.1222); FT-IR (ATR) ν_max_: 3309, 3138, 3073, 2923, 2843, 2051, 1979, 1861, 1683, 1593, 1560, 1518, 1495, 1441, 1350, 1305, 1243, 1189, 1120, 1095, 1042, 979, 931, 829 cm^−1^.

### 3.36. 2-[(1-(4-Fluorophenyl)-triazol-4-yl)methylamino]-naphthalene-1,4-dione (**7r**)

Following the experimental procedure described in method C, from 34.8 mg (0.24 mmol) of 4-fluorophenylboronic acid and 30.0 mg (0.14 mmol) of *N*-propargylated naphthoquinone (**6**), we obtained 21.7 mg (44%) of compound **7r**. ^1^H-NMR (CDCl_3_, 500 MHz) δ 8.10 (1H, m), 8.07 (1H, dd, *J =* 7.6, 1.1 Hz), 7.92 (1H, s), 7.74 (1H, td, *J =* 7.6, 1.3 Hz), 7.70 (2H, m), 7.64 (1H, td, *J =* 7.6, 1.3 Hz), 7.23 (2H, m), 6.40 (1H, s), 5.87 (1H, s), 4.60 (2H, d, *J =* 5.8 Hz) ppm; ^13^C-NMR (CDCl_3_, 125 MHz) δ 183.1 (C), 181.5 (C), 162.6 (C, *J*^1^_C-F_ = 250.3 Hz) 147.5 (C), 143.6 (C), 134.8 (CH), 133.4 (C), 133.1 (C, *J*^4^_C-F_ = 2.6 Hz), 132.2 (CH), 130.5 (C), 126.4 (CH), 126.3 (CH), 122.7 (2 CH, *J^3^*_C-F_ = 8.6 Hz), 120.3 (CH), 116.8 (2 CH, *J*^2^_C-F_ = 23.07 Hz), 101.9 (CH), 38.2 (CH_2_) ppm; EIMS *m*/*z* (%) 348 ([M^+^], 14); 320 (32); 319 (19); 190 (37); 189 (19); 158 (31); 148 (100); 122 (20); 104 (20); 95 (55); 76 (18); 75 (19); HREIMS: 348.1027 (calcd for C_19_H_13_N_4_O_2_F) 348.1023); FT-IR (ATR) ν_max_: 3386, 2364, 2134, 1931, 1683, 1596, 1564, 1500, 1355, 1067, 969, 836 cm^−1^.

### 3.37. 2-[(1-(3-Fluoro-4-methoxyphenyl)-triazol-4-yl)methylamino]-naphthalene-1,4-dione (**7s**)

Following the experimental procedure described in method C, from 41.3 mg (0.24 mmol) of 3-fluoro-4-methoxyphenylboronic acid and 30.0 mg (0.14 mmol) of *N*-propargylated naphthoquinone (**6**), we obtained 24.8 mg (48%) of compound **7s**. ^1^H-NMR (DMSO-*d*_6_, 500 MHz) δ 8.68 (1H, s), 8.02 (2H, m), 7.93 (1H, d, *J =* 7.5 Hz), 7.83 (2H, m), 7.74 (1H, t, *J =* 7.5 Hz), 7.69 (1H, d, *J =* 8.8 Hz), 7.35 (1H, t, *J =* 9.1 Hz), 5.77 (1H, s), 4.57 (2H, d, *J =* 6.1 Hz), 3.89 (3H, s) ppm; ^13^C-NMR (DMSO-*d*_6_, 125 MHz) δ 181.5 (C), 181.3 (C), 151.3 (C, *J^1^*_C-F_ = 246.3 Hz), 148.2 (C), 147.5 (C, *J^2^*_C-F_ = 10.4 Hz), 144.2 (C), 134.7 (CH), 132.9 (C), 132.2 (CH), 130.3 (C), 129.6 (C, *J^3^*_C-F_ = 9.1 Hz), 125.7 (CH), 125.2 (CH), 121.3 (CH), 116.3 (CH, *J^3^*_C-F_ = 3.7 Hz), 114.5 (CH, *J^4^*_C-F_ = 2.7 Hz), 108.7 (CH, *J^2^*_C-F_ = 23.4 Hz), 100.5 (CH), 56.3 (CH_3_), 37.3 (CH_2_) ppm; EIMS *m*/*z* (%): 378 ([M^+^], 23); 350 (19); 349 (12); 179 (11); 178 (100); 84 (11); HREIMS: 378.1132 (calcd for C_20_H_15_N_4_O_3_F) 378.1128); FT-IR (ATR) ν_max_: 3386, 3143, 2235, 2289, 2168, 1683, 1608, 1573, 1533, 1507, 1463, 1354, 1278, 1059, 801 cm^−1^.

### 3.38. 2-[(1-(1-H-Indo-5-yl)-triazol-4-yl)methylamino]-naphthalene-1,4-dione (**7t**)

Following the experimental procedure described in method C, from 39.1 mg (0.24 mmol) of 5-indolylboronic acid and 30.0 mg (0.14 mmol) of *N*-propargylated quinone (**6**), we obtained 22.4 mg (39%) of compound **7t**. ^1^H-NMR (DMSO-*d*_6_, 500 MHz) δ 11.40 (1H, s), 8.65 (1H, s), 8.03 (2H, m), 7.97 (1H, s), 7.93 (1H, d, *J =* 7.6 Hz), 7.82 (1H, t, *J =* 7.5 Hz), 7.74 (1H, t, *J =* 7.5 Hz), 7.58 (2H, s), 7.51 (1H, s), 6.54 (1H, s), 5.81 (1H, s), 4.58 (2H, d, *J =* 6.1 Hz); ^13^C-NMR (DMSO-*d*_6_, 125 MHz) δ 181.5 (C), 181.4 (C), 148.2 (C), 143.7 (C), 135.3 (C), 134.6 (CH), 132.9 (C), 132.1 (CH), 130.4 (C), 129.3 (C), 127.6 (CH), 127.5 (C) 125.7 (CH), 125.2 (CH), 121.5 (CH), 114.1 (CH), 112.1 (CH), 111.8 (CH), 101.8 (CH), 100.4 (CH), 37.40 (CH_2_); EIMS *m/z* (%): 369 ([M^+^], 9); 173 (100); 160 (25); 158 (19); 146 (39); 132 (27); 116 (22); 105 (48); 104 (35); 89 (15); 76 (25); 66 (16); HREIMS: 369.1214 (calcd for C_21_H_15_N_5_O_2_ 369.1226); FT-IR (ATR) ν_max_: 3344, 3274, 2928, 2323, 2254, 2113, 1673, 1604, 1560, 1513, 1458, 1426, 1350, 1309, 1261, 1225, 1123, 1047, 1024, 889, 824 cm^−1^.

### 3.39. Antiplasmodial Assay

F-32 Tanzania (chloroquine-sensitive) strains of *P. falciparum* were cultured according to Trager and Jensen [40] on glucose-enriched RPMI 1640 medium, supplemented with 10% human serum at 37 °C. After 24 h of incubation at 37 °C, the medium was replaced by fresh medium supplemented with the compound to be evaluated at three different concentrations (0.1, 1, and 10 µg/mL) and incubation was continued for a further 48 h. On the third day of the test, a blood smear was taken from each well and parasitemia was calculated for each concentration of sample compared to the control. IC_50_ values were determined graphically by plotting concentrations versus percent inhibition. Chloroquine (0.04 µM) was used as a positive control. All tests were performed in triplicate.

### 3.40. Antiproliferative Activity

The human cancer cell lines HL60 (promyelocytic leukemia), HEL (human erythroleukemia), and SK-Br3 (breast adeno carcinoma) were purchased from ATCC and cultured in RPMI medium 10% FBS. The MTT assay, MTT [3-(4,5-dimethylthiazol-2-yl)-2,5-diphenyl tetrazolium bromide], was used to test the cytotoxicity of 1,2,3-triazolquinones and cell viability [41]. Briefly, cells were plated in 96-well plates at 10,000 cells/well. Sixteen hours after plating, vehicle (0.1% DMSO, final concentration) or compound was added to cells at indicated concentrations. Forty-eight hours following compound addition, MTT (Sigma-Aldrich, St. Louis, MO, USA) was added to each well (0.3 mg/mL, final concentration) and plates were incubated for an additional 2 h at 37 °C. Medium was then aspirated and the formazan product was solubilized in SDS-HCl (20% SDS; HCl 0.02 M). The absorbance of each well was measured at 595 nm using an iMark Microplate Reader (BioRad, Hercules, CA, USA). Nonlinear regression analysis was performed to calculate IC_50_ according to the GraphPad Prism 5 program (GraphPad Software, San Diego, CA, USA). The data were expressed by mean ± SEM (*n* = 3).

### 3.41. Protein Preparation and Docking

The X-ray coordinates of *Plasmodium falciparum* enzyme dihydroorotate dehydrogenase (P*f*DHODH) was extracted from the Protein Data Bank (PDB code 1TV5). The PDB structures were prepared for docking using the Protein Preparation Workflow (Schrodinger, LLC, New York, NY, USA, 2018) accessible from within the Maestro program (Maestro, version 11.5; Schrodinger, LLC: New York, NY, USA, 2018). The substrate and water molecules were removed beyond 5 Å, bond corrections were applied to the cocrystallized ligands, and an exhaustive sampling of the orientations of groups was performed. Finally, the receptors were optimized in Maestro 11.5 by using OPLS3 force field before docking study. In the final stage, the optimization and minimization on the ligand–protein complexes were carried out with the OPLS3 force field and the default value for RMSD of 0.30 Å for non-hydrogen atoms was used. The receptor grids were generated using the prepared proteins, with the docking grids centered on the center of the bound ligand for each receptor. A receptor grid was generated using a 1.00 van der Waals (vdW) radius scaling factor and 0.25 partial charge cutoff. The binding sites were enclosed in a grid box of 20 Å^3^ with default parameters and without constrains. The three-dimensional structures of the ligands to be docked were generated and prepared using LigPrep as implemented in Maestro 11.5 (LigPrep, Schrodinger, LLC: New York, NY, USA, 2018) to generate the most probable ionization states at pH 7 ± 1 (retain original ionization state). These conformations were used as the initial input structures for the docking. In this stage, a series of treatments are applied to the structures. Finally, the geometries are optimized using OPLS3 force field. These conformations were used as the initial input structures for the docking. The ligands were docked using the extra precision mode (XP) [42] without using any constraints and a 0.80 van der Waals (vdW) radius scaling factor and 0.15 partial charge cutoff. The dockings were carried out with flexibility of the residues of the pocket near to the ligand. The generated ligand poses were evaluated with empirical scoring function, GlideScore a modified version of ChemScore [43]; GlideScore implemented in Glide was used to estimate binding affinity and rank ligands [44]. The XP Pose Rank was used to select the best-docked pose for each ligand.

## 4. Conclusions

In summay, a library of 34 1,2,3-triazolyl naphthoquinone derivatives was prepared using a copper (I) catalyzed Huisgen 1,3-dipolar cycloaddition reaction of *O*-propargylated naphthoquinone (**1**) or *N*-propargylated naphthoquinone (**6**) and different azides. Some simplified analogues were also synthesized in order to deepen the structural determinants responsible of the antiplasmodial activity. The compounds were evaluated against strains of *Plasmodium falciparum* F-32 Tanzania (chloroquine sensitive). The results showed that the best antiplasmodial activities were achieved with the derivatives having the ether bridge and aromatic substituents attached at the nitrogen of the triazole ring. The nature of the substituents on the aromatic ring seemed to modulate the antiplasmodial activity. The results obtained from docking studies supported the hypothesis that the enzyme P*f*DHODH might be the target of these compounds in the parasite. The most active compounds (**3d**, **3f**, **3j**, **3l**, **3m**, and **3s**) showed values for physicochemical descriptors within the accepted ranges for drug-like molecules. All these results suggest that these compounds could serve as promising lead compounds for further research.

## Figures and Tables

**Table 1:** Synthesis and structure of 1,2,3-triazole-naphthoquinones (**3a–3u**).

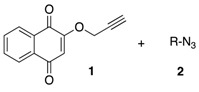	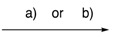	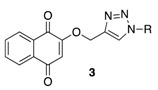
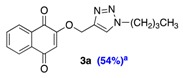	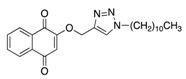	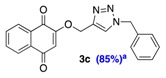
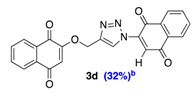	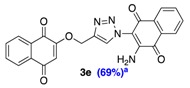	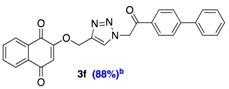
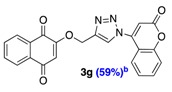	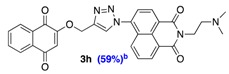	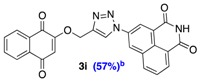
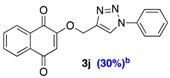	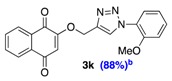	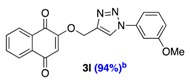
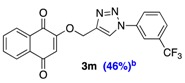	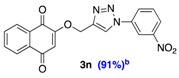	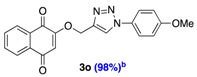
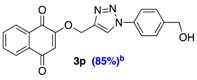	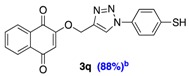	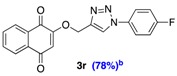
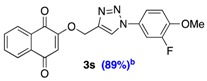	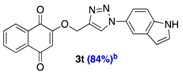	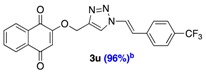

^a^ CuSO_4_·5H_2_O, sodium ascorbate, CH_2_Cl_2_/H_2_O (1:1); ^b^ CuI, CH_3_CN.

**Table 2:** In vitro activity of compounds (**3a**–**3u**) against *Plasmodium falciparum* F32 Tanzania (IC_50_ μM) and against SkBr3 (human breast cancer), MCF-7 (human breast adenocarcinoma), and HEL (human erythroleukemia) cell lines (IC_50_ μM).

Compound	*P. falciparum*	SkBr3	MCF-7	HEL
**3a**	11.2 ± 0.3	8.0 ± 0.9	7.6 ± 1.0	4.6 ± 0.9
**3b**	17.0 ± 0.7	>10	>10	7.3 ± 0.6
**3c**	17.9 ± 2.5	>10	>10	5.9 ± 0.7
**3d**	1.9 ± 0.09	>10	>10	>10
**3e**	>20	>10	>10	5.6 ± 0.6
**3f**	1.6 ± 0.1	>10	>10	7.00 ± 1.0
**3g**	>20	>10	>10	>10
**3h**	>20	5.7 ± 0.8	4.9 ± 0.8	4.9 ± 0.7
**3i**	5.5 ± 0.1	>10	>10	5.5 ± 1.0
**3j**	1.2 ± 0.2	>10	>10	>10
**3k**	4.4 ± 2.5	>10	>10	>10
**3l**	1.7 ± 0.1	>10	>10	>10
**3m**	1.7 ± 0.09	>10	>10	>10
**3n**	2.4 ± 0.2	>10	>10	>10
**3o**	2.2 ± 0.1	>10	>10	>10
**3p**	9.7 ± 0.2	>10	6.5 ± 0.9	3.9 ± 0.8
**3q**	>20	>10	>10	>10
**3r**	2.0 ± 0.09	>10	>10	3.8 ± 0.6
**3s**	0.8 ± 0.1	>10	>10	>10
**3t**	>20	>10	>10	>10
**3u**	3.3 ± 0.07	>10	>10	>10
**chloroquine**	0.15 ± 0.02	----	----	----
**adriamycin**	----	0.1 ± 0.06	0.6 ± 0.09	0.1 ± 0.03

**Table 3:** Preparation of isosteric analogues of 1,2,3-triazole-naphthoquinones (**7b–7d**, **7h**, **7j–7o**, **7r–7t**).

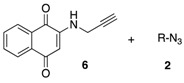	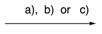	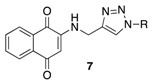
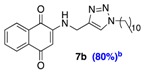	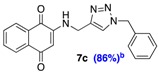	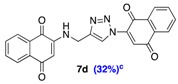
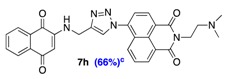	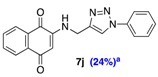	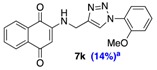
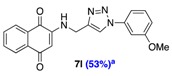	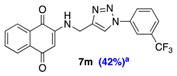	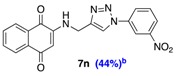
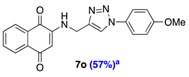	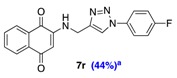	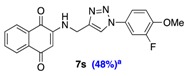
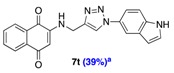		

^a^ Cu_2_O, sodium ascorbate, acetone/H_2_O; ^b^ CuSO_4_·5H_2_O, sodium ascorbate, CH_2_Cl_2_/H_2_O (1:1); ^c^ CuI, THF.

**Table 4:** Physicochemical descriptors of **3d**, **3f**, **3j**, **3l**, **3m,** and **3s**. ^a,b^

Compound	MW	LogP	H-Bond Donors	H-Bond Acceptors	Rotable Bonds	TPSA
**3d**	411	2.76	0	8	4	108.24
**3f**	449	4.34	0	7	7	91.17
**3j**	331	2.68	0	6	4	74.09
**3l**	361	2.92	0	7	5	83.33
**3m**	399	3.76	0	6	5	74.09
**3s**	379	3.04	0	7	5	83.33

^a^ Values were calculated using Molinspiration Cheminformatics software (Molinspiration, Slovensky Grob, Slovak Republic, 2019, http://www.molinspiration.com); ^b^ optimal range MW < 500, LogP < 5, H-bond donors < 5, H-bond acceptors < 10, Rotable bonds < 5, TPSA < 140.

## Supplementary Materials

The following are available online: Synthesis of alkynes **1** and **6**, azides and phenyl triazole derivatives (**5j**, **5l**, **5n,** and **5o**). ^1^H-NMR and ^13^C-NMR spectra of compounds **3a–3u**, **4a**, **4j**, **5j**, **5l**, **5n**, **5o**, **7b–7d**, **7h, 7j–7o, 7s–7t**.
